# HBI: a hierarchical Bayesian interaction model to estimate cell-type-specific methylation quantitative trait loci incorporating priors from cell-sorted bisulfite sequencing data

**DOI:** 10.1186/s13059-024-03411-7

**Published:** 2024-10-15

**Authors:** Youshu Cheng, Biao Cai, Hongyu Li, Xinyu Zhang, Gypsyamber D’Souza, Sadeep Shrestha, Andrew Edmonds, Jacquelyn Meyers, Margaret Fischl, Seble Kassaye, Kathryn Anastos, Mardge Cohen, Bradley E. Aouizerat, Ke Xu, Hongyu Zhao

**Affiliations:** 1 Department of Biostatistics, Yale School of Public Health, New Haven, CT 06511 USA; 2 VA Connecticut Healthcare System, West Haven, CT 06516 USA; 3 Department of Psychiatry, Yale School of Medicine, New Haven, CT 06511 USA; 4 Department of Epidemiology, Johns Hopkins Bloomberg School of Public Health, Baltimore, MD USA; 5 Department of Epidemiology, School of Public Health, University of Alabama at Birmingham, Birmingham, AL 35294 USA; 6 The University of North Carolina at Chapel Hill, Chapel Hill, NC USA; 7 Department of Psychiatry, SUNY Downstate Health Sciences University School of Medicine, Brooklyn, NY USA; 8 Department of Medicine, University of Miami School of Medicine, Miami, FL USA; 9 Division of Infectious Diseases and Tropical Medicine, Georgetown University, Washington, DC USA; 10 Department of Medicine, Albert Einstein College of Medicine, New York, NY USA; 11 Hektoen Institute for Medical Research, Chicago, IL USA; 12 Bluestone Center for Clinical Research, College of Dentistry, New York University, New York, NY USA; 13 Department of Oral and Maxillofacial Surgery, College of Dentistry, New York University, New York, NY USA

**Keywords:** Methylation quantitative trait loci, Cell-type-specific DNA methylation, hierarchical Bayesian interaction model, Cell-sorted methylation sequencing data, Colocalization

## Abstract

Methylation quantitative trait loci (meQTLs) quantify the effects of genetic variants on DNA methylation levels. However, most published studies utilize bulk methylation datasets composed of different cell types and limit our understanding of cell-type-specific methylation regulation. We propose a hierarchical Bayesian interaction (HBI) model to infer cell-type-specific meQTLs, which integrates a large-scale bulk methylation data and a small-scale cell-type-specific methylation data. Through simulations, we show that HBI enhances the estimation of cell-type-specific meQTLs. In real data analyses, we demonstrate that HBI can further improve the functional annotation of genetic variants and identify biologically relevant cell types for complex traits.

**Supplementary Information:**

The online version contains supplementary material available at 10.1186/s13059-024-03411-7.

## Background

DNA methylation (DNAm) is one of the most widely studied epigenetic modifications that capture the cumulative effects of environmental and genetic factors. DNAm regulates cellular differentiation and gene expression and plays a key role in human development and disease etiology [1, 2]. Single-nucleotide polymorphisms (SNPs) associated with DNAm levels are known as methylation quantitative trait loci (meQTLs) [3–6], which capture and represent the complex interplay between the genome and methylome.

To reveal cellular mechanisms for DNAm patterns and their link to complex traits, it is important to study cell-type-specific (CTS) genetic effects on DNAm (CTS-meQTL). For example, SNP rs174548, which is mapped on *FADS1*, a key enzyme in the metabolism of fatty acids, is associated with asthma [1]. At the same time, its effect on methylation at cg21709803 is the strongest in CD8+ T-cells. These results suggest a possible effect of rs174548 on asthma via immune dysregulation and fatty acid metabolism through methylation in CD8+ T-cells [1]. However, most meQTL studies to date have used bulk samples composed of distinct cell types [7–9]. MeQTLs identified from bulk DNAm samples reflect the aggregated genetic effects across all cell types, which provide no insights for genetic regulations in individual cell types. This approach is especially problematic for rare or less abundant cell types. The high cost and technical limitations for both cell sorting and single-cell DNAm approaches hinder the collection of large-scale, CTS methylation profiles, and limit our ability to move meQTL studies from the “bulk level” to the “cell type level.”

Given the difficulty in generating large-scale CTS methylome data to directly estimate CTS effects and the broad availability of many bulk methylation datasets, several statistical methods have been developed to infer CTS meQTLs from bulk data. These methods can be classified into two categories. Methods in the first category estimate sample-level CTS DNAm profiles from bulk data in the first step, and then test the associations with outcomes of interest using the deconvoluted data for each cell type. Tensor Composition Analysis (TCA), a frequentist approach in this category, was originally designed to identify CTS differentially methylated CpG sites in epigenome-wide association studies of phenotypes (CTS-EWAS) [10]. There is also a similar algorithm designed for gene expression data [11], named Bayesian MIND (bMIND), which further incorporates information from single-cell RNA sequencing (scRNA-seq) data as a prior to refine the estimation of CTS expression for each bulk sample. bMIND innovatively integrates large-scale bulk data and small-scale CTS expression data from scRNA-seq to estimate CTS expression for large-scale bulk samples. In contrast, methods in the second category are based on an interaction model to test the interaction between cell type fractions and variables of interest without deconvolution. Examples include CellDMC [12], which focuses on the interaction between cell type fractions and phenotypes (CTS-EWAS). Westra et al. also proposed an interaction model to estimate CTS expression quantitative trait loci (CTS-eQTL) [13].

Here we introduce a hierarchical Bayesian interaction model (HBI) to infer CTS meQTLs from bulk methylation data. Our model allows the incorporation of cell-type-specific DNAm data from a relatively small number of samples to improve the performance of HBI. Compared with bMIND, which utilizes Bayesian techniques to infer the posterior mean of sample-level CTS expression (or as easily for methylation), the goal of HBI is instead to infer the posterior mean of CTS genetic effects by placing sparse hierarchical priors on regression coefficients for the interaction terms. In our model, we employ hierarchical double-exponential priors to induce different shrinkage for different variables, which corresponds to the Bayesian adaptive lasso [14]. If cell-type-specific data are available for a small number of samples (e.g., 5–10% of the sample size in bulk data), the algorithm can incorporate this information to further refine the estimates for CTS genetic effects in the larger-scale bulk samples. In our case, cell-sorted Methylation Capture sequencing data (MC-seq) is used to derive CTS DNAm and since it offers the unique advantage of directly measuring CTS methylomes, incorporating strong and robust signals from the MC-seq data will improve the estimation of CTS-meQTLs.

We show in simulations that HBI improves the estimation of CTS genetic effects when compared to other state-of-the-art methods [10–12]. We apply our method to identify cis CTS-meQTL using data from samples in the Women’s Interagency HIV Study (WIHS) (*n*_bulk_ = 431, *n*_CTS_ = 47), now the MACS/WIHS Combined Cohort Study (MWCCS) [15]. To demonstrate the utility of our method, we use an independent meQTL dataset derived from CTS methylation data [1] to evaluate the replication of HBI-identified signals. Finally, we perform downstream analyses to improve the annotation of functional genetic variants and to reveal the cellular specificity of complex traits.

## Results

### Estimation of CTS-meQTLs using HBI

**Fig. 1:**
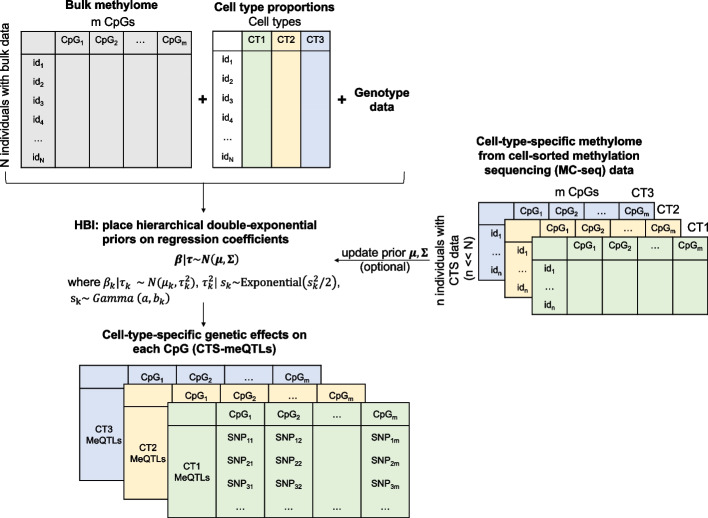
Overview of the hierarchical Bayesian interaction model (HBI) to infer cell type-specific (CTS) meQTLs. With bulk methylation data and cell type proportions (we present an example of three cell types: CT1, CT2, CT3), HBI employs an interaction model with sparse hierarchical priors placed on the regression coefficients for the interaction terms. If the CTS DNA methylation data (in our case, generated by methylation capture-sequencing using cells sorted from PBMC using flow cytometry) are available for a small group of samples, HBI will further incorporate the information into priors to refine the estimates for CTS genetic effects in the larger-scale bulk samples

A linear regression framework including interaction terms between genotype/phenotype and estimated cell type fractions has been applied to identify CTS-QTL or CTS- differentially methylated CpGs [12, 13]. Here, based on this idea, we propose HBI to incorporate prior information from CTS DNAm data and to improve the estimation of CTS-meQTLs (Fig. 1). We place hierarchical double-exponential priors on regression coefficients for the interaction terms:

$$\beta_k\vert\tau_k^2\sim N(\mu_k,\tau_k^2),$$

$$\tau_k^2\vert s_k\sim\text{Exp}\left(\frac{s_k^2}{2}\right),$$

where ${\beta }_{k}$ is the regression coefficient on the interaction term between genotype and cell type proportion for the $k$ th cell type. Marginalizing over ${\tau }_{k}^{2}$, ${\beta }_{k}$ conditional on ${s}_{k}$ follows a double-exponential distribution:

$${\beta }_{k}|{s}_{k}\sim DE({\mu }_{k}, 1/{s}_{k}),$$

where parameter ${s}_{k}$ controls the degree of shrinkage. If ${s}_{k}$ is a fixed value for $k=\text{1,2},\dots ,K$, each variable will be shrunk to the same degree. Here we model ${s}_{k}$ as a hyperparameter to allow for variable-specific penalty:

$${s}_{k}\sim \text{Gamma}\left(\text{a},{\text{b}}_{\text{k}}\right),$$

where $a$ and ${b}_{k}$ are chosen based on empirical experiments (Methods).

In the case where only bulk data are available, we set ${\mu }_{k}=0$ for $k=\text{1,2},\dots ,K$ and the model would be similar to the adaptive Lasso approach [14, 16]: the regression coefficients for interaction terms are shrunk to 0 and the degree of shrinkage differs for different variables. Such shrinkage helps to take the sparsity of genetic effects into consideration. When the CTS methylomes are available for a small number of samples, we can first get a rough estimate of the genetic effect in the $k$ th cell type ${\widehat{\beta }}_{k,seq}$ using the small set. Then instead of setting ${\mu }_{k}=0$ and shrinking the coefficient to zero, we can shrink it to a more meaningful value by updating the prior mean ${\mu }_{k}:$

$$\mu_\kappa=weight\cdot{\widehat\beta}_{k,seq}+\left(1-weight\right)\cdot0,$$

where ${\mu }_{k}$ is a weighted sum between ${\widehat{\beta }}_{k,seq}$ (observed results from CTS methylomes) and zero (prior beliefs), while ${weight}={1-p}_{adjust}$ and ${p}_{adjust}$ is the *p*-value adjusted using the Bonferroni correction (Methods). Similar to other studies that propose weights based on posterior probabilities [17], the weights in our model are assigned based on *p*-values as *p*-value is a probability measuring the evidence against the null hypothesis (${\beta }_{k,seq}=0$) [18] and can reflect the stability of the estimator ${\widehat{\beta }}_{k,seq}$. Intuitively, a small *p*-value closer to zero indicates ${\widehat{\beta }}_{k,seq}$ estimated using the CTS DNAm data is relatively strong. In this case, we shrink the coefficient more towards ${\mu }_{k}= {\widehat{\beta }}_{k,seq}$. In contrast, a large *p*-value closer to one indicates ${\widehat{\beta }}_{k,seq}$ is not significantly different from zero, and thus we shrink it more towards ${\mu }_{k}= 0$.

Along with updating prior means, we can also update prior variances:

$${\varvec{\beta}}|{\varvec{\tau}}\sim {\varvec{N}}\left({\varvec{\mu}}=\boldsymbol{ }\left[\begin{array}{c}{ \mu }_{1}\\ {\mu }_{2}\\ \vdots \\ {\mu }_{K}\end{array}\right],\boldsymbol{ }{\varvec{\Sigma}}=\boldsymbol{ }\boldsymbol{ }\left[\begin{array}{cccc}{\tau }_{1}^{2}& {\rho }_{12}{\tau }_{1}{\tau }_{2}& \cdots & {\rho }_{1K}{\tau }_{1}{\tau }_{K}\\ {\rho }_{12}{\tau }_{2}{\tau }_{1}& {\tau }_{2}^{2}& \cdots & {\rho }_{2K}{\tau }_{2}{\tau }_{K}\\ \vdots & \vdots & \ddots & \vdots \\ {\rho }_{1K}{\tau }_{K}{\tau }_{1}& {\rho }_{2K}{\tau }_{K}{\tau }_{2}& \cdots & {\tau }_{K}^{2}\end{array}\right]\right),$$

where ${\rho }_{jk}$ can be updated as the genetic correlation between cell type $k$ methylation and cell type $j$ methylation, which can also be estimated from the CTS methylomes provided. The prior variance without CTS data can be seen as a special case with all ${\rho }_{jk}=0$. Of note, as the detection of genetic effects is always much harder in less abundant cell types, the incorporation of the estimated genetic correlation aims to improve the power in the less abundant cell types by borrowing information from the more abundant cell types.

More details of our model are summarized in “Methods”. We note the following key features for HBI: (1) because only a few SNPs among hundreds of SNPs surrounding a CpG may have detectable effects, placing a sharp prior centered at 0 helps to take the sparsity of genetic effects into consideration; (2) local shrinkage parameters ${s}_{k}$ and ${\tau }_{k}^{2}$ make the algorithm more flexible: the degree of shrinkage could differ among variables; and (3) priors could be updated to incorporate information from CTS DNAm data, if they are available for a small group of samples.

### HBI improves performance in simulations

We evaluated the performance of HBI in estimating CTS-meQTLs through extensive simulations. We considered three scenarios: (1) there are genetic effects only in the major/most abundant cell type; (2) there are genetic effects only in the minor/least abundant cell type; and (3) there are correlated genetic effects in all cell types. We compared HBI to other state-of-the-art methods: bMIND, TCA, and the basic interaction model fitted by ordinary least squares (OLS) (Methods). In each scenario, we assessed the correlation between the estimated and true effect sizes, mean squared error (MSE) between the estimated and true effect sizes, power, and false discovery rate (FDR) as a function of the proportion of causal SNPs.

**Fig. 2:**
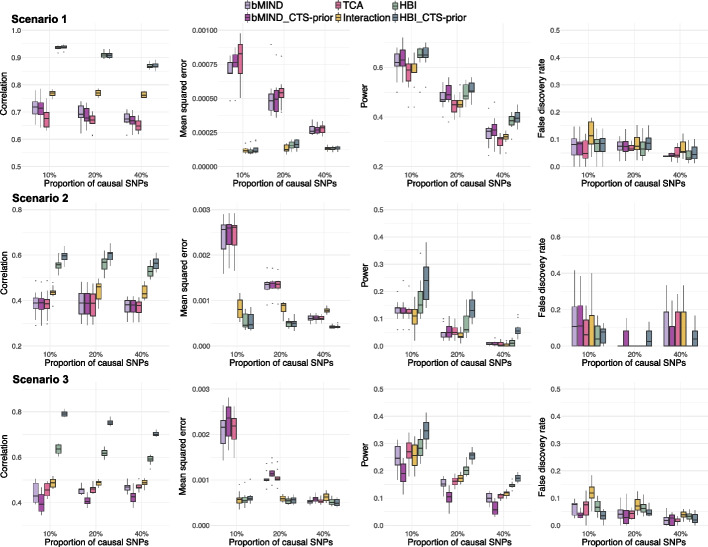
Comparisons of performance in estimating cell type-specific (CTS)-meQTLs. From top to bottom: scenarios with genetic effects only in the most abundant cell type (Scenario 1), only in the least abundant cell type (Scenario 2), and with correlated genetic effects in all cell types (Scenario 3) are shown. From left to right: correlation between estimated and true effect sizes, mean squared error (MSE) between estimated and true effect sizes, power, and false discovery rate (FDR) as a function of the proportion of causal SNPs. HBI_CTS-prior, bMIND_CTS-prior represent the version of the corresponding methods with CTS DNA methylation data incorporated

HBI improved the point estimation of CTS-meQTLs by achieving higher correlation and lower MSE (Fig. 2). For example, in scenario 1 when the proportion of causal SNPs fixed at 10%, the median of correlation across 10 simulations was 0.72 for bMIND with only bulk data (denoted as “bMIND”), 0.71 for bMIND with CTS data incorporated (denoted as “bMIND_CTS-prior”), 0.68 for TCA, 0.77 for basic interaction model, 0.94 for HBI with only bulk data (denoted as “HBI”), and 0.94 for HBI with CTS data incorporated (denoted as “HBI_CTS-prior”). Across all scenarios, HBI generally achieved higher power compared with other methods. We note that in scenario 1, when genetic effects only occur in the most abundant cell type, further incorporating CTS DNAm data to update priors had comparable power to the case without incorporating CTS DNAm data. For example, in scenario 1 when the proportion of causal SNPs fixed at 10%, the median of power across 10 simulations was both 0.65 for HBI with only bulk data (denoted as “HBI”) and HBI with CTS data incorporated (denoted as “HBI_CTS-prior”). In contrast, in scenarios 2 and 3, when there were genetic effects in the minor/least abundant cell type, incorporating information from CTS DNAm data helped to improve the power. For example, in scenario 2 when the proportion of causal SNPs fixed at 10%, the median of power across 10 simulations was 0.15 for HBI with only bulk data (denoted as “HBI”), and 0.24 for HBI with CTS data incorporated (denoted as “HBI_CTS-prior”). In each scenario, we varied the proportion of causal SNPs from 10 to 20% to 40%, to compare the performance of these methods when the genetic effects became more polygenic. As expected, the power for all methods decreased as the proportion of causal SNPs increased. When the overall genetic effect (heritability) was fixed and diluted on a larger number of SNPs, it generally became harder to detect signals. We also note that the performance for bMIND and TCA was a result of fitting conditional models (Methods). In the case of fitting marginal models for bMIND and TCA, we observed inflated FDR, especially in scenarios 1 and 2 when there were genetic effects only in one single cell type (Additional file 1: Fig S1).

Of note, all methods included here relied on cell type proportions, but in reality the biological “ground truth” of the cell type proportions is rarely available and the computationally estimated proportions [19, 20] are used directly, which introduces additional noise. Therefore, to further evaluate the robustness of all methods when “noisy” cell type proportions (random error was added to the true proportions) were given, we repeated the simulation scenario 3 but with noisy proportions inputted for all methods (Methods). With the increase in the noise added to cell type proportions, the correlation and power decreased while the MSE increased (Additional file 1: Fig S2), as expected. HBI was robust in this “noisy” setting by achieving the highest correlation and power and lowest MSE among all the methods considered. Additional results comparing the performance across different allele frequencies and different numbers of SNP-CpG pairs are summarized in Additional file 1: Fig S3-4.

To further investigate the performance of HBI in real data for which we also have estimates of the “ground truth”, we applied those methods to the Religious Orders Study and Memory and Aging Project (ROSMAP) gene expression and genotype data and the “ground truth” for QTLs was estimated with its single-cell RNA seq data [21]. We noted that HBI can also be used to identify CTS expression quantitative trait loci (eQTL). Similar patterns were observed, where HBI achieved higher power across different scenarios (Additional file 1: Fig S5).

### Genome-wide CTS-meQTLs identification using HBI

To identify genome-wide CTS-meQTLs, we applied HBI to the WIHS cohort with matched genotype data and bulk DNAm data measured in peripheral blood mononuclear cells (PBMC) using the Illumina HumanMethylation EPIC beadchip (*n* = 431) (Additional file 2: Table S1). Furthermore, for a separate group of WIHS participants (*n* = 47), one aliquot of PBMC underwent CTS separation to obtain CD4+ T-cells (*n* = 28), CD8+ T-cells (*n* = 28), or monocytes (*n* = 27). The demographic characteristics of the WIHS participants are described in Additional file 2: Table S1. DNAm from each sorted cell type was profiled using Agilent SureSelectXT Methyl-seq, and the derived priors from these CTS DNAm data were incorporated in HBI (Methods). Significant *cis*-meQTLs were selected as those reaching genome-wide significance level (*p* < 6E − 12; Bonferroni correction). The computational time for applying HBI is summarized in Additional file 1: Fig S6, and the median computational time was about 4 min.

**Fig. 3:**
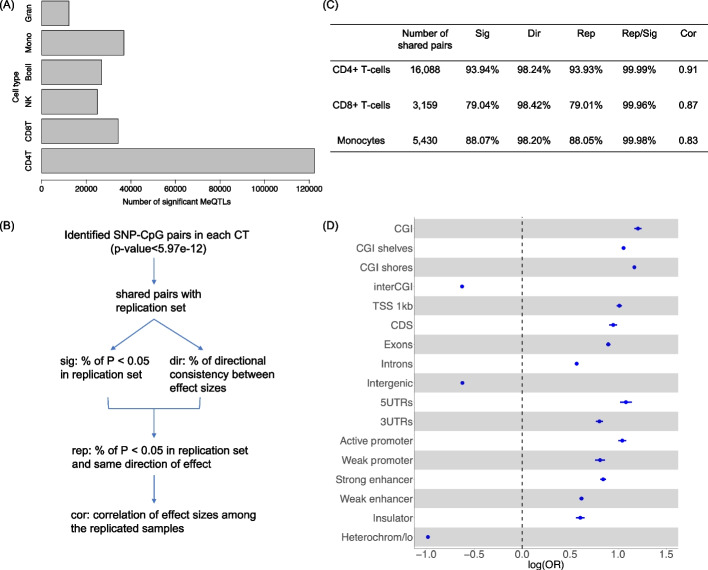
Overview of cell type-specific (CTS)-meQTLs identification using the hierarchical Bayesian interaction model (HBI).** A **Bar chart shows the number of HBI-identified meQTLs in each cell type (*p*<6E−12). **B** Flow chart indicates the replication of HBI identified CTS-meQTLs in an independent dataset for meQTLs in isolated white blood cell subsets (CD4+ T-cells, CD8+ T-cells, monocytes). **C** Table summarizes the replication results. **D** Functional enrichment for meQTLs across all cell types in CpG island (CGI) regions, gene body regions, and gene regulatory regions. The logarithm of odds ratio (OR) with 95% confidence interval is presented. TSS 1 kb: <1 kb upstream of the transcription start site (TSS); CDS: coding sequence; UTR: untranslated exon region; Heterochrom/lo: regions that exhibit heterochromatic or heterochromatin-like characteristics; CD4T: CD4+ T-cells; CD8T: CD8+ T-cells; NK: natural killer cells; Mono: monocytes; Gran: granulocytes

HBI identified a total of 122,387 significant meQTLs in CD4+ T-cells, 34,310 in CD8+ T-cells, 25,020 in natural killer cells, 26,972 in B cells, 36,919 in monocytes, and 12,231 in granulocytes (Fig. 3A) (Additional file 3: Table S2). To replicate our identified CTS-meQTLs, we used publicly available data for meQTLs in isolated white blood cell subsets (CD4+ T-cells, CD8+ T-cells, monocytes) (*n* = 60 individuals) [1], and we defined replicated meQTLs as those with *p* < 0.05 and consistent direction of effect in this replication sample (Fig. 3B). We showed that among the shared SNP-CpG pairs in the replication sample, 98.2–98.4% had a consistent direction of effect and 79.0–93.9% were replicated (Fig. 3C). Of note, in all cell types, more than 99% of significant pairs (*p* < 0.05) had a consistent direction of effect (Rep/Sig), indicating a high level of consistency between our results in the WIHS sample and those in the replication sample. We also investigated the replication rates using the version of HBI without priors incorporated from the WIHS participants with CTS data (*n* = 47), and did parallel analyses using SNPs in high LD to increase the number of shared pairs in the replication sample (Additional file 4: Table S3). An additional data with larger sample sizes for meQTLs in isolated blood cells (CD4+ T-cells, monocytes) (*n* = 197 individuals) was also used for replication [22]. The similar pattern was observed for replication rates of HBI (97.31% in CD4+ T-cells, 92.40% in monocytes) (Additional file 4: Table S3).

Integrating annotations of CpG islands (CGI), genomic functional regions, and open chromatin states with our derived CTS-meQTL, we observed that compared with SNPs that are not meQTLs (non-meQTLs), our identified meQTLs across all cell types were enriched in important regulatory regions, such as active promotors and strong enhancers (Fig. 3D). Conversely, our meQTLs were depleted in regions with few active genes, including intergenic regions and regions with heterochromatic characteristics, as previously reported [7]. Of note, meQTLs identified in each cell type exhibited similar functional enrichment patterns and are summarized in Additional file 1: Fig S7.

Using QIAGEN Ingenuity Pathway Analysis (IPA) to perform pathway enrichment analyses of genes mapped by the significant meQTLs in each cell type [23], we found that the antigen presentation pathway was significant in multiple cell types: CD4+ T-cells (*p* = 2.95E − 05), CD8+ T-cells (*p* = 1.12E − 09), B cells (*p* = 9.55E − 06), natural killer cells (*p* = 7.94E − 07) and monocytes (*p* = 1.41E − 10) (Additional file 5: Table S4). Other identified pathways included the pulmonary fibrosis idiopathic signaling pathway in CD4+ T-cells (*p* = 9.33E − 06), the multiple sclerosis signaling pathway and the IL-15 production pathway in CD8+ T-cells (*p* = 9.55E − 07 and *p* = 3.63E − 05, respectively). These significant pathways indicated that the identified CTS-meQTLs by HBI might play a role in regulating immunity-related functions and activities.

### CTS meQTLs colocalize with risk variants for complex traits

While for most meQTLs the direct impact on complex traits has not been widely reported [24], there have been studies showing that some meQTLs are associated with complex traits and may identify underlying pathways and mechanisms related to diseases [7, 8, 25]. To systematically identify potential associations between meQTLs and complex traits, we applied HyPrColoc (Hypothesis Prioritization for multi-trait Colocalization) [26] to perform an meQTL-GWAS colocalization analysis in each cell type. We integrated the HBI-identified CTS-meQTLs with 57 GWAS datasets in four categories of blood cell counts, cardiometabolic, immune, and allergy [27].

**Fig. 4:**
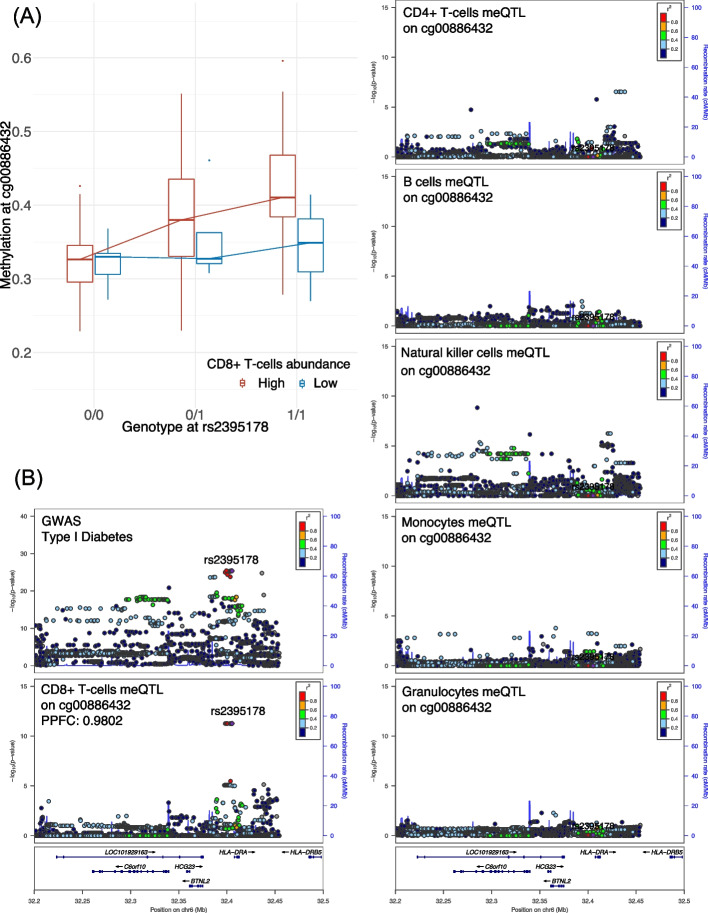
Example of the rs2395178-cg00886432 locus and colocalization results with type I diabetes (T1D).** A** An association plot for the rs2395178-cg00886432 locus, separated into individuals with high and low abundance of CD8+ T-cells (above and below the median, respectively). The *y* axis shows methylation beta-values, while the *x* axis shows genotypes. **B** LocusZoom plots for the association of rs2395178 (mapped to *HLA-DRA*) with phenotypes/molecular traits. Panels illustrate the association of the SNP with GWAS T1D, cg00886432 meQTL signal in CD8+ T-cells, CD4+ T-cells, B cells, natural killer cells, monocytes, and granulocytes. The genetic variant rs2395178 was identified as a colocalized SNP between T1D and cg00886432 meQTL signal in CD8+ T-cells (posterior probability for colocalization (PPFC) is shown)

A total of 2972 significant meQTL-GWAS colocalizations (posterior probability for colocalization (PPFC) > 0.50) were identified across all GWASs and cell types (Additional file 6: Table S5A-F). Taking a further look into the number of meQTL-GWAS colocalizations per trait across all cell types, we found that GWAS traits in the category of blood cell counts had a larger number of colocalizations compared with traits in other categories (Additional file 1: Fig S8). This abundance of colocalizations was expected as the *cis*-meQTLs were identified in cell types from whole blood. To further illustrate how the meQTL-GWAS colocalizations could differ across cell types, we summarize one example in Fig. 4. The variant rs2395178 in the *HLA-DRA* gene was identified as a CD8+ T-cell-specific meQTL for cg00886432 (*p* = 5.46E − 12). As expected, we observed that rs2395178 showed a stronger correlation with DNAm in participants with a high abundance of CD8+ T-cells (Fig. 4A). Meanwhile, our colocalization analyses revealed that rs2395178 was colocalized between methylation at cg00886432 in CD8+ T-cells and type I diabetes (T1D) (PPFC = 0.9802) (Fig. 4B), while no significant colocalization was observed in other cell types. Of note, polymorphisms at the *HLA-DQ* and *HLA-DR* regions have been recognized as the major genetic determinants of T1D [28]. Taken together, these results suggest that integrating DNA methylome and genome data may help link *HLA-DR* gene function in CD8+ T-cells to T1D.

### MeQTL-GWAS colocalizations exhibit enrichment in trait-relevant cell types

To further investigate meQTL associations with traits in multiple cell types, we performed enrichment analyses to study if the meQTL-GWAS colocalizations for each trait were enriched in certain cell types. Specifically, for each trait we defined the enrichment score in one cell type as the ratio between the percentage of colocalized GWAS-meQTLs in that cell type and the percentage of meQTLs in that cell type (see Methods). As the absolute number of colocalizations in each cell type was largely driven by the number of identified meQTLs in that cell type and cannot be compared directly, here the enrichment score was defined as the ratio between two percentages, which allowed us to compare this value across different cell types. We further excluded granulocytes due to the low number of colocalizations identified across traits (Additional file 1: Fig S8B), which indicated the less stable signals identified in this least abundant cell-type. We also evaluated the enrichment score for meQTLs derived at the bulk PBMC level (Additional file 6: Table S5G) to further evaluate whether CTS-meQTLs can reveal more cell-specific information.

**Fig. 5:**
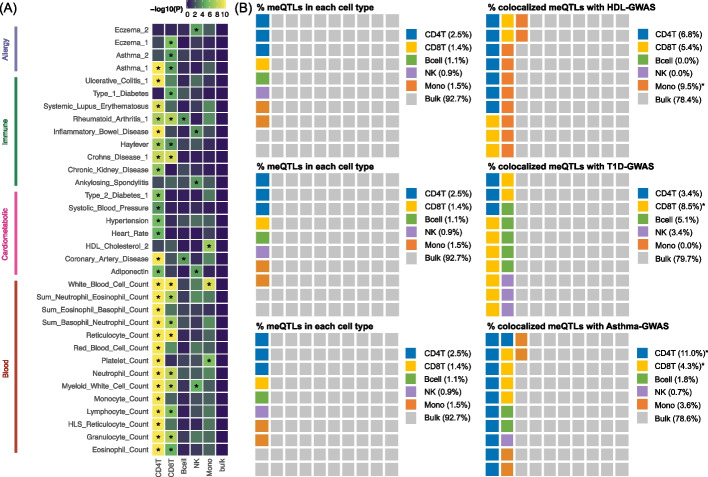
Enrichment analyses for MeQTL-GWAS colocalizations. **A **Colocalization enrichment results across six cell types for traits with colocalizations enriched in at least one cell type. Asterisks highlight significance after Bonferroni correction. **B** Examples of colocalization enrichments in three traits. From left to right: the percentage of meQTLs covered by each cell type, and the percentage of colocalized meQTLs in that cell type. GWAS: genome-wide association studies; CD4T: CD4+ T-cells; CD8T: CD8+ T-cells; NK: natural killer cells; Mono: monocytes; bulk: a mixture of cell types from peripheral blood mononuclear cells (PBMC)

We summarize the traits with colocalizations enriched in at least one cell type in Fig. 5A, which we listed out in Additional file 7: Table S6A. To evaluate whether the enrichment results matched existing biological knowledge, we performed heritability enrichment analyses across the same GWAS traits using GenoSkyline-Plus [29], which could be viewed as an independent tool to identify biologically relevant cell types for complex traits. We found that our significant findings generally agreed with the results of heritability enrichment analyses: 85.2% of our identified cell types with enriched colocalizations were replicated by GenoSkyline-Plus (Additional file 7: Table S6B). This indicates that the colocalizations between HBI-identified CTS-meQTLs and GWASs do help to reveal biologically relevant cell types for complex traits. For example, high-density lipoprotein cholesterol (HDL) had colocalization enrichment in monocytes: monocytes only covered 1.5% of the total meQTLs but accounted for 9.5% of the colocalized meQTLs (enrichment = 6.32; *p* = 1.23E − 07) (Fig. 5B). Of note, GenoSkyline-Plus also identified heritability enrichment in monocytes for HDL (*p* = 1.31E − 06) (Additional file 7: Table S6B). In T1D, we identified colocalization enrichment in CD8+ T-cells (enrichment = 6.15; *p* = 1.95E − 05) (Fig. 5B), while the heritability for this trait was also enriched in CD8+ T-cells (*p* = 4.77E − 02) (Additional file 7: Table S6B). This finding is further supported by the evidence that autoreactive CD8+ T-cells play a fundamental role in the progression of T1D by the destruction of pancreatic beta cells [30]. In addition, asthma was enriched in colocalizations derived from CD4+ T-cells (enrichment = 4.34; *p* = 5.14E − 19) and CD8+ T-cells (enrichment = 3.10; *p* = 4.91E − 05) meQTLs, which was also replicated by GenoSkyline-Plus (*p* = 3.43E − 03 and *p* = 3.92E − 05, respectively) (Additional file 7: Table S6B). Interestingly, the association between meQTLs and asthma has been investigated by Hawe et al., who also employed colocalization and reported a shared causal variant rs174548 for methylation at cg21709803 in CD8+ T-cells and asthma [1]. Here our colocalization results in CD8+ T-cells replicated and extended their findings by identifying a nearby risk variant rs174587 (PPFC = 0.858) (Additional file 6: Table S5B), which impacts both DNAm at cg21709803 and asthma.

From Fig. 5B, we show that more bulk meQTLs were identified than CTS meQTLs, which was consistent with simulations (Additional file 1: Fig S9), but we observed no colocalization enrichments (Fig. 5A). This suggests that the meQTLs identified in bulk tissue are a mixture of signals from different cell types, thus masking the CTS information. Altogether, those results suggest that our identified CTS meQTLs can provide more insight into the cellular specificity of complex traits and aid the characterization of trait etiology.

## Discussion

We have developed HBI to infer CTS meQTLs from bulk methylation data, with priors derived from CTS methylation data in a small group of samples. As far as we are aware, our model is the first one to integrate large-scale bulk DNAm data and small-scale CTS DNAm data to estimate CTS-meQTLs. We show through simulations that HBI improves the estimation of CTS genetic effects. Applying our method to samples contributed by participants from the WIHS cohort [15], we systematically characterized the genome-wide SNP-CpG associations in multiple cell types of PBMCs. Through colocalization and enrichment analyses, we demonstrate the utility of HBI to improve the annotation of functional genetic variants and enhance the understanding of the cellular specificity of complex traits.

We considered extensive simulation scenarios to compare the performance of different methods in detecting CTS QTLs. As TCA and bMIND were initially developed to detect differentially expressed or differentially methylated signals between comparison groups (e.g., cases versus controls) [10, 11], the differential effect was on a single phenotype of interest in their simulations. In contrast, in our simulations the genetic effects on a CpG were distributed across a number of SNPs and each SNP carried a small effect. Therefore, our simulation aims to detect all causal SNPs, which is more challenging than detecting the association with one single phenotype, and the simulation results may offer a more comprehensive evaluation of the performance of different methods to detect CTS-QTLs than those considered in other studies [10, 11, 31].

The simulation results show that all methods had decreased performance in scenario 2 (the least abundant cell type harbored genetic effects). This is not surprising as the information from rare cell types is in general more limited in a bulk sample, and thus the statistical instability for estimating signals in rare cell types is much larger than that in abundant cell types. In this case, incorporating CTS DNAm information did help to alleviate this problem; we show that HBI with CTS information incorporated into priors (i.e., HBI_CTS-prior) was more powerful than other methods. Specifically, to improve the power to infer meQTL in rare cell types by borrowing information from more abundant cell types, we used the small group with CTS methylation data to estimate ${\rho }_{jk}$, the genetic correlation between cell type $k$ methylation and cell type $j$ methylation, and incorporated the estimated genetic correlation into the prior variance. As cell-sorted MC-seq data offer the unique advantage of directly measuring CTS methylomes, incorporating strong and robust signals from such data improves the estimation of CTS-meQTLs, especially in rare cell types.

We observed inflated FDR when fitting the marginal model for bMIND and TCA. As discussed by the authors of TCA, deconvoluted CTS methylation profiles are expected to be correlated among different cell types [32], which results in false discoveries in the non-causal cell type when using the marginal test model. To mitigate this problem, the TCA authors advised applying a marginal conditional test to account for the other cell types [32], which was also used in our simulations. The developers of bMIND also proposed an alternative testing procedure, in which they only detected the top cell type with the minimal differential expressed (DE) *p*-value within a gene. This testing procedure was supported by some single-nucleus RNA-sequencing (snRNA-Seq) studies [33, 34] which reported that most CTS DE genes are only differentially expressed in one single cell type. In contrast, our meQTL analyses aimed to capture not only meQTLs that are specific in one single cell type but also meQTLs that are shared across multiple or all cell types. Previous studies have reported the existence of a substantial proportion of meQTLs that exhibit this shared pattern across diverse cell types [1, 35]. Therefore, in our simulations, we did not adopt the alternative testing procedure proposed by bMIND. Instead, the marginal conditional test model was fitted for TCA and bMIND to control FDR and to provide a fair comparison with HBI. Although HBI generally outperformed other methods in our QTL-based simulations, we note that the deconvolution step in TCA and bMIND can output sample-level CTS profiles which enable other sample-level analyses (e.g., CTS co-expression networks), while methods based on the interaction model, like HBI, do not have this additional output.

In real data applications, the use of stringent statistical thresholds and independent replication datasets [1] enables the identification of CTS-meQTLs with high confidence and generalizability. Specifically, our identified meQTLs were supported by high replication rates in isolated CD4+ T-cells, CD8+ T-cells, and monocytes. We highlighted one example of the potential of our approach to identify biologically relevant cell types for complex traits. The colocalization analyses between meQTLs and GWASs for T1D identified several SNPs in the HLA region. For example, rs2395178 in *HLA-DRA* was identified as a CD8+ T-cell specific meQTL for cg00886432. *HLA-DRA* belongs to the human leukocyte antigen (HLA) complex family, which plays an important role in antigen presentation and immune defense [36], and is well known as the major genetic determinant of T1D [28]. Our colocalization analyses revealed that rs2395178 was shared between methylation at cg00886432 in CD8+ T-cells and T1D (PPFC = 0.9802). There has also been evidence for the contribution of CD8+ T-cells to the progression of T1D by the destruction of pancreatic beta cells [30]. Altogether, our downstream analyses helped to explain the relationship between this SNP-CpG locus and T1D, especially in CD8+ T-cells. We also noted that the colocalized signals might be driven by haplotype structures or LD, as multiple studies identified strong signals for T1D at nearby SNPs in the HLA region (e.g., rs9271365 mapped to *HLA-DQA1*) [37–39], which are close to but not identical to the colocalized SNPs that we identified. Similarly, for other complex traits, we also identified biologically relevant cell types through meQTL-GWAS colocalization, and our findings strongly agreed with heritability enrichment analyses [29]. We also investigated the computational time of HBI in this real data application. The computational time increased linearly with the number of SNP-CpG pairs in one CpG. The median of pairs in one CpG was 1624 and the median of computational time was 4.05 min.

We acknowledge several limitations of our study. First, similar to other methods [10–12], our model depends on cell type proportions ${W}_{k}$ as an input. Currently, the method described by Houseman et al. [19, 40] was widely applied to estimate this ${W}_{k}$ for blood samples. There are also some efforts to quantify ${W}_{k}$ for non-blood samples (i.e., brain samples) [41]. However, those estimated proportions are used directly to approximate the biological ground truth, which introduces additional error. For example, we identified significant meQTLs for granulocytes, which theoretically should have been filtered out in PBMC. This may result from technical issues including granulocyte contamination during PBMC processing [42], and the inaccuracy in the estimated granulocyte proportions. To alleviate this issue, we plan to extend the statistical model to estimate the cell type proportions and incorporate the uncertainty in the estimated proportions at the same time. This approach will broaden the applications as we will not rely on other algorithms to estimate cell type proportions, and help to obtain more accurate results as the uncertainty in the estimated proportions is considered and adjusted. Second, we used an meQTL dataset obtained from experimentally isolated white blood cells [1] as the “gold standard” to replicate our findings. However, their CpG data were generated using the Illumina Human Methylation 450 K BeadChip while our results were based on the Illumina Infinium Methylation EPIC BeadChip. Additionally, only a total of 11.2 million SNP-CpG pairs that were preselected in their bulk meQTL analysis were available. As a result, not all our significant results were represented in their database, even though we utilized SNPs in high LD to increase the number of shared pairs. Third, in colocalization enrichment analysis, we did not observe significant results for some traits (i.e., no significant enrichments for heart attack or stroke). The potential reasons might be that our CTS-meQTLs were only derived from white blood cells and may not cover the causal cell types, and the small number of identified colocalizations in some traits may impact our results as well. Therefore, re-applying HBI on a dataset with a larger sample size and a wider range of causal cell types will help to obtain a more powered and complete CTS-meQTL catalog [35].

## Conclusions

HBI provides a statistical strategy to leverage bulk data and CTS MC-seq data to improve the estimation of CTS meQTLs. Through in-depth real data analyses, we linked the methylome and genome data and illustrated the power of HBI to identify biologically relevant cell types for complex traits. We believe that HBI can have wide applications in identifying CTS meQTLs and annotating functional genetic variants.

## Methods

### Statistical model

We model the relationship between methylation level at one CpG and one SNP as:

1 $$\text{M} = \sum\limits_{\text{c}=1}^{\text{C}}\upgamma_{\text{c}}\text{X}_{\text{c}} + \sum\limits_{\text{k}=1}^{\text{K}}\upalpha_{\text{k}}\text{W}_{\text{k}} + \sum\limits_{\text{k}=1}^{\text{K}}\upbeta_{\text{k}}\left(\text{W}_{\text{k}}\cdot \text{G}\right) + \upepsilon,$$

where $M$ is the bulk methylation level, ${W}_{k}$ is the proportion of the $k$ th cell type, $G$ is the genotype of the SNP (the number of alternative alleles) of interest, ${X}_{c}$ represents the $c$ th covariate, such as age, sex, or ancestry, $\epsilon$ is a normally distributed error, and ${\alpha }_{k}, {\beta }_{k}, {\gamma }_{c}$ are regression coefficients. The coefficients of the interaction terms ${\beta }_{k}$ are of primary interest: intuitively, if there exist genetic effects of DNAm in cell type $k$, the observed association between methylation and genotype should be stronger in samples with a higher fraction of cell type $k$ compared to samples with lower fractions [12]. Of note, this model without intercept is equivalent to the following one, due to the fact that cell type proportions add up to 1:

2 $$\text{M} = {\sum\limits_{\text{c} = 1}^{\text{C}}{\widetilde{\upgamma}}_{\text{c}}{\textrm{X}}_{\text{c}} + {\widetilde{\upalpha}}_{0}} + \sum\limits_{\text{k} = 1}^{\text{K}-1}{\widetilde{\upalpha}}_{\text{k}}{\textrm{W}}_{\text{k}} +{\widetilde{\upbeta}}_{0}\text{G} + \sum\limits_{\text{k}=1}^{\text{K}-1}{\widetilde{\upbeta}}_{\text{k}}\left(\text{W}_{\textrm{k}}\cdot \text{G}\right)+ \upepsilon .$$

The difference between the two models lies in the interpretation of coefficients. In model (1), ${\beta }_{k}$ represents the genetic effects on DNAm in cell type $k$ ($k=\text{1,2},\dots ,K$). In model (2), ${\widetilde{\beta }}_{0}$ represents the genetic effects on DNAm in cell type $K$ and ${\widetilde{\beta }}_{k}$ represents the changes in genetic effects in cell type $k$ ($k=\text{1,2},\dots ,K-1$) compared to the effect ${\widetilde{\beta }}_{0}$ in cell type $K$. Therefore, ${\widetilde{\beta }}_{0}+{\widetilde{\beta }}_{k}$ corresponds to the genetic effects in cell type $k$ ($k=\text{1,2},\dots ,K-1$). For simplicity, we use model (1) in the following derivations.

In order to take the sparsity of genetic effects into consideration and to update information derived from CTS methylation data from a small group of samples, a hierarchical framework is used to construct priors for regression coefficients [43]. To achieve optimal performance, we recommend that the small group of samples with CTS methylation data can be a subset of the overall samples with bulk data, or be similar samples drawn from the same study or cohort as the bulk samples. We first assume a multivariate normal distribution for coefficients for interaction terms ${{\varvec{\beta}}}$:

$${\varvec{\beta}}\left|{\varvec{\tau}}=\left[\begin{array}{c}{\beta }_{1}\\ {\beta }_{2}\\ \vdots \\ {\beta }_{K}\end{array}\right]\right|{\varvec{\tau}}\sim {\varvec{N}}\left({\varvec{\mu}},\boldsymbol{ }{\varvec{\Sigma}}\right)$$

- with general prior (no CTS methylation data)
  3 $$\begin{array}{c}\mu =0, \Sigma = \left[\begin{array}{cccc}{\tau }_{1}^{2}& 0& \cdots & 0\\ 0& {\tau }_{2}^{2}& \cdots & 0\\ \vdots & \vdots & \ddots & \vdots \\ 0& 0& \cdots & {\tau }_{K}^{2}\end{array}\right]\end{array}$$

- with prior derived from CTS methylation data of a small group of samples
  4 $$\begin{array}{c}\mu = \left[\begin{array}{c}{ \mu }_{1}\\ {\mu }_{2}\\ \vdots \\ {\mu }_{K}\end{array}\right], \Sigma = \left[\begin{array}{cccc}{\tau }_{1}^{2}& {\rho }_{12}{\tau }_{1}{\tau }_{2}& \cdots & {\rho }_{1K}{\tau }_{1}{\tau }_{K}\\ {\rho }_{12}{\tau }_{2}{\tau }_{1}& {\tau }_{2}^{2}& \cdots & {\rho }_{2K}{\tau }_{2}{\tau }_{K}\\ \vdots & \vdots & \ddots & \vdots \\ {\rho }_{1K}{\tau }_{K}{\tau }_{1}& {\rho }_{2K}{\tau }_{K}{\tau }_{2}& \cdots & {\tau }_{K}^{2}\end{array}\right]\end{array}$$

where ${\mu }_{k}$ and ${\rho }_{jk}$ are updated from the CTS methylation data in the following ways:

1. ${\mu }_{k}$ = ${w}_{\mu }\cdot {\widehat{\beta }}_{k,seq}+{(1-w}_{\mu })\cdot 0$, where ${\widehat{\beta }}_{k,seq}$ is the estimated effect when regressing the cell type $k$ methylome on the SNP, and ${w}_{\mu }={1-p}_{adjust}$ where ${p}_{adjust}$ is the *p*-value adjusted by Benjamini & Hochberg (BH) or Bonferroni [17], as defined by users.
2. ${\rho }_{jk}= {w}_{\rho }\cdot {\widehat{\rho }}_{jk,seq}+{(1-w}_{\rho })\cdot 0$, where ${\widehat{\rho }}_{jk,seq}$ is the estimated genetic correlation between cell type $k$ methylation and cell type $j$ methylation, and ${w}_{\rho }={1-p}_{adjust}$ where ${p}_{adjust}$ is the corresponding *p*-value for ${\widehat{\rho }}_{jk,seq}$, adjusted by Benjamini & Hochberg (BH) or Bonferroni [17], as defined by users.

In both settings with and without CTS methylomes derived from CTS DNAm data, the variable-specific parameter ${\tau }_{k}^{2}$ controls the degree of shrinkage: as ${\tau }_{k}^{2}$ gets close to 0, ${\beta }_{k}$ is shrunk to ${\mu }_{k}$, while as ${\tau }_{k}^{2}$ gets larger, the amount of shrinkage will be smaller. We further model ${\tau }_{k}^{2}$ using the exponential distribution with variable-specific hyperparameters ${s}_{k}$:

5 $$\begin{array}{c}{\tau }_{k}^{2}|{s}_{k}\sim Exp\left(\frac{{s}_{k}^{2}}{2}\right), \end{array}$$

where ${s}_{k}$ was modelled using a gamma distribution as a hyper-prior:

6 $$\begin{array}{c}{s}_{k}\sim Gamma\left(\text{a},{\text{b}}_{\text{k}}\right).\end{array}$$

In this way, we allow different degrees of shrinkage for different variables by introducing the variable-specific parameters ${s}_{k}$ and ${\tau }_{k}^{2}$. We also derive the conditional posterior distributions of ${s}_{k}$ and ${\tau }_{k}^{2}$ as follows:

7 $$\begin{array}{c}{s}_{k}|{\beta }_{k}\sim Gamma\left(\text{a}+1,{\text{b}}_{\text{k}}+\left|{\beta }_{k}-{\upmu }_{\text{k}}\right|\right),\end{array}$$

8 $$\begin{array}{c}1/{\tau }_{k}^{2}|{s}_{k},{\beta }_{k}\sim Inverse\,Gaussian \left(\frac{{s}_{k}}{\left|{\upbeta }_{\text{k}}-{\upmu }_{\text{k}}\right|}, {s}_{k}^{2}\right).\end{array}$$

These will be used in the model fitting algorithm.

### EM-IWLS algorithm for model fitting and inference

We fit the hierarchical Bayesian interaction model by a modified iterative weighted least squares (IWLS) algorithm, proposed by Yi et al. [43]. Compared with usual IWLS, the new method incorporates an expectation–maximization (EM) algorithm that treats the unknown variances ${\tau }_{k}^{2}$ and the hyperparameter ${s}_{k}$ as missing data and estimates the ${{\varvec{\beta}}}$ by averaging over these missing values; hence, it is also referred to as the EM-IWLS algorithm.

In each iteration of the E-step, we update the missing values $({s}_{k}, {\tau}_{k}^{2})$ by their conditional expectations derived from (7) and (8). In the M-step, we update ${{\varvec{\beta}}}$ by maximizing the expected log-likelihood. We need to incorporate the prior ${\varvec{\beta}}|{\varvec{\tau}}$ into the normal likelihood as additional data points [44]. Let $J$ denote the total number of variables: ($J-K$) covariates (e.g., ${\alpha }_{k}, {\gamma }_{c}$) included to address potential confounding, and $K$ covariates (${\varvec{\beta}}$) of our interest, and let ${{\varvec{\theta}}}={\left[{{\varvec{\gamma}}}^{{\varvec{T}}},\boldsymbol{ }{\boldsymbol{\alpha }}^{{\varvec{T}}},{{\varvec{\beta}}}^{{\varvec{T}}}\right]}^{{\varvec{T}}}\in {{\varvec{R}}}^{J}$. Model (1) could be expressed as:

9 $$\begin{array}{c}M= X\theta +\epsilon ,\end{array}$$

where ${\varvec{X}}\in {{\varvec{R}}}^{n\times J}$ is the original design matrix in model (1).

Then we update the regression coefficients by running the augmented linear regression:

10 $$\begin{array}{c}{{\varvec{y}}}_{\boldsymbol{*}}\sim N\left({{\varvec{X}}}_{\boldsymbol{*}}{\varvec{\theta}},\phi {{\varvec{\Sigma}}}_{\mathbf{*}}\right),\end{array}$$

where ${{\varvec{y}}}_{\boldsymbol{*}}^{\boldsymbol{ }}={\left[{{\varvec{M}}}^{{\varvec{T}}},\boldsymbol{ }{0}^{{\varvec{T}}},\boldsymbol{ }{{\varvec{\mu}}}^{{\varvec{T}}}\boldsymbol{ }\right]}^{{\varvec{T}}}$ is an ($\left(n+J\right)\times 1$) vector of methylation levels for $n$ samples and prior means for $J$ covariates, $${{\varvec{X}}}_{\boldsymbol{*}}=\boldsymbol{ }\left[\begin{array}{c}{\varvec{X}}\\ {{\varvec{I}}}_{{\varvec{J}}}\end{array}\right]$$ is an ($\left(n+J\right)\times J$) matrix constructed by the design matrix ${\varvec{X}}$ in (9) and the identity matrix, and $${{\varvec{\Sigma}}}_{\mathbf{*}}=\boldsymbol{ }\left[\begin{array}{ccc}{{\varvec{I}}}_{{\varvec{n}}}& 0& 0\\ 0& \frac{1}{{\varvec{\phi}}}{{\varvec{\Sigma}}}_{22}& 0\\ 0& 0& \frac{1}{{\varvec{\phi}}}{{\varvec{\Sigma}}}_{33}\end{array}\right]$$ is an ($\left(n+J\right)\times \left(n+J\right)$) matrix with ${{\varvec{\Sigma}}}_{22}=$ diag(${\tau }_{1}^{2}, {\tau }_{2}^{2}, \dots ,{\tau }_{\left(J-K\right)}^{2}$) and ${{\varvec{\Sigma}}}_{33}=\boldsymbol{ }{{\varvec{\Sigma}}}$ is the ($K\times K$) prior variance matrix for ${\varvec{\beta}}|{\varvec{\tau}}$**.** Then in each iteration, we can update the estimates:

11 $$\begin{array}{c}\widehat{{\varvec{\theta}}}={\left({{\varvec{X}}}_{\boldsymbol{*}}^{{\varvec{T}}}{{\varvec{\Sigma}}}_{\mathbf{*}}^{-1}{{\varvec{X}}}_{\boldsymbol{*}}\right)}^{-1}{{\varvec{X}}}_{\boldsymbol{*}}^{{\varvec{T}}}{{\varvec{\Sigma}}}_{\mathbf{*}}^{-1}{{\varvec{y}}}_{\boldsymbol{*}}^{\boldsymbol{ }},\end{array}$$

12 $$\begin{array}{c}\widehat{\phi }=\frac{1}{{\varvec{n}}}{\left({{\varvec{y}}}_{\boldsymbol{*}}-{{\varvec{X}}}_{\boldsymbol{*}}\widehat{{\varvec{\theta}}}\right)}^{{\varvec{T}}}{{\varvec{\Sigma}}}_{\mathbf{*}}^{-1}{\left({{\varvec{y}}}_{\boldsymbol{*}}-{{\varvec{X}}}_{\boldsymbol{*}}\widehat{{\varvec{\theta}}}\right)}^{\boldsymbol{ }},\end{array}$$

until convergence. We can also get the variance of regression coefficients:

13 $$\begin{array}{c}var\left(\widehat{{\varvec{\theta}}}\right)={\left({{\varvec{X}}}_{\boldsymbol{*}}^{{\varvec{T}}}{{\varvec{\Sigma}}}_{\mathbf{*}}^{-1}{{\varvec{X}}}_{\boldsymbol{*}}\right)}^{-1}\widehat{\phi }.\end{array}$$

The EM-IWLS algorithm is summarized as follows. 
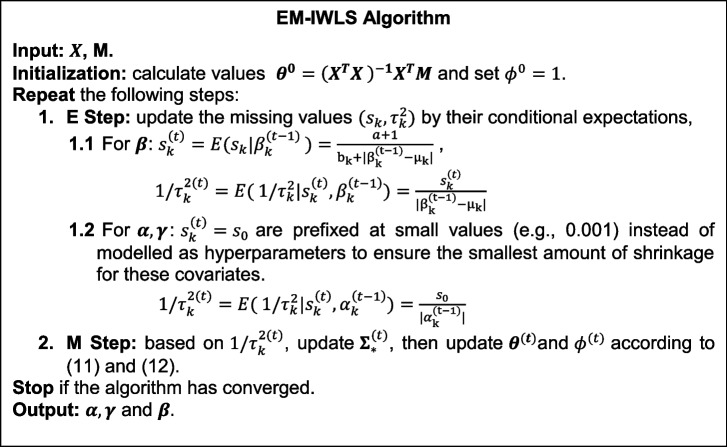


We define convergence as each element of ${|{\varvec{\theta}}}^{\left({\varvec{t}}\right)}-{{\varvec{\theta}}}^{\left({\varvec{t}}-1\right)}|$ smaller than $\delta$, with $\delta$ to be a small value (e.g., 1E − 05). $\widehat{{\varvec{\theta}}}$ and ${\varvec{v}}{\varvec{a}}{\varvec{r}}\left(\widehat{{\varvec{\theta}}}\right)$ can then be obtained from the last updates.

For the choice of $\left(\text{a},{\text{b}}_{\text{k}}\right)$, we fix $\text{a}=0.5$ as the default since the overall degree of shrinkage can be determined by ${\text{b}}_{\text{k}}$ [43]. For the user-defined ${\text{b}}_{\text{k}}$, we suggest taking the sample size and the abundance of the corresponding cell type into consideration. For moderate sample size (e.g., $\text{n}=O\left({10}^{2}\right)$), we suggest ${\text{b}}_{\text{k}}=0.2$ for most cell types (average of cell type proportions > 10%), and ${\text{b}}_{\text{k}}=5$ to induce a less informative prior for the least abundant cell type (average of cell type proportions < 5%). Otherwise, the estimation of the coefficient for the least abundant cell type might be overwhelmingly driven by the prior. For larger sample sizes (e.g., $n=O\left({10}^{4}\right)$) or much more abundant cell type, ${\text{b}}_{\text{k}}$ could be decreased accordingly.

### Simulation settings

In this section, we introduce the simulation procedure to evaluate the performance of HBI. CTS DNAm in our simulations were generated based on genotype data from the Wellcome Trust Case Control Consortium (WTCCC) (*n* = 15,918) [45]. In the cell type with genetic effects, the heritability of the DNAm was fixed as 0.3, the effect sizes of the causal SNPs were generated by a multivariate normal distribution [46], and GCTA [47] was applied to simulate the DNAm in this cell type. We also generated cell type proportions using a Dirichlet distribution for three cell types with parameters 5.30, 1.27, and 1.62. These parameters were chosen based on the suggestion from Li et al. that the mean cell type composition standard deviation is around 0.13, which was estimated from the Cibersort blood true proportions [48]. Then, for each sample, the bulk DNAm levels were computed as a weighted sum of the simulated CTS DNAm levels, weighted by the corresponding cell type proportions, plus an independent and identically distributed (iid) noise term $\epsilon \sim N\left(0, 0.01\right)$.

We considered three main scenarios, and in each of them, we assumed that the total number of SNPs near the simulated CpG site to be 500 and varied the proportion of causal SNPs from 10% to 20% to 40%. All the SNPs were randomly selected from chromosome 12 and all have minor allele frequency (MAF) > 0.01. To investigate whether variants with lower frequency have low power and high false positives, we further divided the SNPs into variants with low frequency (0.01 ≤ MAF < 0.05) and common variants (MAF ≥ 0.05), and assessed their performance separately as supplementary results. Each simulation setting was repeated 10 times.

1. Scenario 1: there were genetic effects only in the major/most abundant cell type.
2. Scenario 2: there were genetic effects only in the minor/least abundant cell type.
3. Scenario 3: there were correlated genetic effects in all three cell types, and the genetic correlation among the cell types was set to 0.5.

We compared our method HBI with TCA [10], bMIND [11], and the basic interaction model, which fits model (1) directly using OLS and is similar to the CellDMC algorithm [12]. For HBI and the basic interaction model, we inputted the simulated bulk DNAm and cell type proportions and directly obtained the genetic effects for each cell type as the estimated coefficients for the interaction terms (${W}_{k}\cdot G$). The choices of the HBI parameters in the hyper prior $Gamma\left(\text{a},{\text{b}}_{\text{k}}\right)$ were as follows: $\text{a}=0.5$ for all cell types, ${\text{b}}_{\text{k}}=0.005$ for the major cell type, ${\text{b}}_{\text{k}}=0.1$ for the other two cell types. For TCA and bMIND, we first inputted the bulk DNAm and cell type proportions to get deconvoluted CTS DNAm, and then tested the association between CTS DNAm and genotype using PLINK [49] to fit the following two models:

1. Marginal model which regresses the deconvoluted DNAm for cell $j$, $\widehat{{Z}_{j}}$, on the genotype $G$ (equivalent to *marginal test* in TCA) [32]:
  $$\widehat{{Z}_{j}} \sim G.$$
2. Conditional model which regresses the deconvoluted DNAm for cell $j$ on the genotype with DNAm for all other cell types controlled (equivalent to *marginal conditional test* in TCA):
  $$\widehat{{Z}_{j}}\sim G+ \sum\nolimits_{l\ne j}\widehat{{Z}_{l}}.$$

The coefficients for genotype $G$ would then be the estimated genetic effects in cell $j$. CTS-meQTLs were identified with FDR controlled at 0.05 for each cell type. In each setting, the performance of different methods was compared in terms of correlation between the estimated and true effect sizes, the MSE between the estimated and true effect sizes, power, and FDR calculated as follows:

$$power= \frac{\#\,identified\,true\,signals\,in\,all\,cell\,types}{\#\,true\,signals\,in\,all\,cell\,types},$$

$$false\,discovery\,rate= \frac{\#\,identified\,false\,signals\,in\,all\,cell\,types}{\#\,identified\,signals\,in\,all\,cell\,types}$$

Both HBI and bMIND had the optional step to incorporate CTS information to update priors (derived from cell-sorted MC-seq data in our case and from scRNA-seq data in bMIND’s original case). Here we also assumed that for a small proportion (5%) of all samples, their CTS methylation data were available. In each simulation setting, we further compared HBI and bMIND both without this information incorporated and with this information incorporated (denoted as HBI_CTS-prior, bMIND_CTS-prior).

Since all methods included here relied on cell type proportions, we further evaluated the robustness of all methods when noisy cell type proportions were given. With the proportion of causal SNPs fixed as 20% in scenario 3, we randomly simulated noise from a left-truncated normal distribution (truncation point is zero), added noise to the true cell type proportions, and then normalized the sum of proportions to be 1. Two additional simulation settings were performed as we adjusted the standard deviation of the added noise so that the generated noisy cell type proportions would have mean absolute error (MAE) of 0.05 and 0.1, respectively. In addition, to assess the effect of the number of SNPs near the simulated CpG site, we performed additional simulations and varied the number of total SNPs from 500, 1000, to 2000, with the proportion of causal SNPs fixed as 10%.

To further investigate the simulation performance of HBI using real data, we utilized the samples in ROSMAP data with matched gene expression and genotype [21] (*n* = 290). We first estimated the “ground truth” using its single-cell RNA seq data. We included three cell types: excitatory neurons, inhibitory neurons, and oligodendrocytes, and estimated their eQTLs separately. We then extracted the significant eQTLs (Bonferroni-adjusted *p* < 0.05) fitting into 3 scenarios: (1) eQTLs only in excitatory neurons (simulated as the major cell type in pseudo-bulk), (2) eQTLs only in oligodendrocytes (simulated as the minor cell type in pseudo-bulk), and (3) eQTLs in all three cell types. The effect sizes of those eQTLs estimated by single-cell RNA seq data were treated as “ground truth”. Pseudo-bulk data consisting of the 3 cell types were then created as the input for TCA, bMIND, the basic interaction model, and HBI. In each repeat, we randomly sampled 500 eQTLs in each scenario and applied all the methods to evaluate their power to correctly identify those eQTLs. Similarly, the performance was compared in terms of correlation between the estimated and true effect sizes, the MSE between the estimated and true effect sizes, power, and FDR.

### Study cohort for real data applications

The Women’s Interagency HIV Study (WIHS), now a part of MWCCS, is a multi-center, prospective, observational cohort study [15]. All participants are women with HIV or at risk for HIV acquisition. Informed consent was provided by all WIHS participants via protocols approved by institutional review committees at each affiliated institution. In our analysis, participants with matched genetic data and bulk DNA methylation measured in PBMC (*n* = 431) and a separate group of participants with CTS DNA methylation data (*n* = 47) were included. Demographic and clinical characteristics are summarized in Additional file 2: Table S1.

### Genotyping, imputation, and quality control

The WIHS sample were genotyped using the Infinium Omni2.5 Bead-Chip that targeted approximately 2.4 million SNPs. Minimac4 was used for imputation with the 1000 Genomes Project 3 as the reference panel [50, 51]. We removed SNPs with minor allele frequency < 0.05, missing rate > 5%, imputation quality *r*^2^ < 0.8, and those that deviated significantly from Hardy–Weinberg equilibrium (*p* < 1e − 6). As a result, approximately 4.6 million SNPs passed QC and were used for CTS-meQTL estimation.

### DNA methylation

DNA methylation measured using DNA isolated from PBMC was profiled using the Illumina Infinium MethylationEPIC BeadChip. We followed methods described in Lehne et al. [52] to perform methylation normalization and adjust for potential batch effects. A total of 852,073 CpGs for the 431 individuals passed quality control steps and were used as bulk DNAm data. We applied the method described by Houseman et al. to estimate the cell type proportions for CD4+ T-cells, CD8+ T-cells, natural killer cells, B cells, monocytes, and granulocytes [19, 40]. Another separate group of the WIHS cohort (*n* = 47) were isolated for CD4+ T-cells, CD8+ T-cells, and monocytes. DNAm for each sorted cell type was profiled by the Agilent SureSelectXT Methyl-seq. After quality control and extracting CpGs that overlapped on both platforms, we had 390,851 CpGs measured in CD4+ T-cells (*n* = 28), 385,679 CpGs measured in CD8+ T-cells (*n* = 28), and 407,646 CpGs measured in monocytes (*n* = 27), which were used as CTS DNAm data to update priors.

### CTS-meQTL estimation and replication

We applied HBI to identify CTS meQTLs in the WIHS cohort for six cell types: CD4+ T-cells, CD8+ T-cells, natural killer cells, B cells, monocytes, and granulocytes. For each CpG, we considered the following model for SNPs from 500 kb upstream to 500 kb downstream [53–56]:

$$M=\sum_{C=1}^C\gamma_cX_c+\sum_{k=1}^6\alpha_kW_k+\sum_{k=1}^6\beta_k\left(W_k\cdot G\right),$$

where $M$ is the bulk methylation M-value, ${W}_{k}$ is the cell type proportion of the $k$ th cell type, $G$ is the genotype of the SNP, ${X}_{c}$ is a collection of previously identified relevant covariates: age, estimated global ancestry, local ancestry [55], tobacco use, alcohol consumption, HIV infection status, log_10_ of HIV RNA viral load, the top 5 genotype principal components (PCs), and the top 10 PCs on DNA methylation levels of control probe. HBI was applied to estimate the regression coefficients in the above model, and for CD4+ T-cells, CD8+ T-cells, and monocytes, we further incorporated the priors derived from the CTS methylation data available in a small group of subjects. The choices of the parameters in the hyper prior $Gamma\left(\text{a},{\text{b}}_{\text{k}}\right)$ were as follows: $\text{a}=0.5$ for all cell types, ${\text{b}}_{\text{k}}=5$ for granulocytes, ${\text{b}}_{\text{k}}=0.2$ for natural killer cells, B cells, monocytes, and ${\text{b}}_{\text{k}}=0.05$ for CD4+ T-cells, CD8+ T-cells. Among the 852,073 CpGs, a total of 1.4 billion SNP-CpG pairs were tested, and significant meQTLs were selected using Bonferroni correction (*p* < 0.05/1,384,706,562/6 = 6.02E − 12). Due to the low proportion of granulocytes, we also conducted a sensitivity analysis with five-cell-type decomposition (proportion of granulocytes removed). CpGs on chromosome 22 were used in the sensitivity analysis and results are summarized in Additional file 1: Fig S10.

Independent data were used to replicate our identified CTS-meQTLs. We downloaded datasets for meQTLs in isolated white blood cell subsets (i.e., CD4+ T-cells, CD8+ T-cells, monocytes, neutrophils) (*n* = 60 individuals) [1]. For our identified SNP-CpG pairs in the respective cell types, we calculated the percentage of pairs that were significant in the replication set (*p* < 0.05), the percentage of pairs with directional consistency in effect sizes, and the percentage of replicated pairs (*p* < 0.05 and same effect direction). Among the replicated pairs, we also calculated the correlations of the effect sizes. Considering the limited sample size for this dataset (*n* = 60) [1], we also included another data with larger sample sizes for meQTLs in isolated blood cells (CD4+ T-cells, monocytes) (*n* = 197) [22] as supplementary results for replication.

To investigate the replication rates of the version of HBI with only bulk data, we conducted parallel analyses using HBI without priors incorporated from the WIHS participants with CTS data (*n* = 47). In addition, to increase the number of shared SNP-CpG pairs between our results and the replication data, we further utilized SNPs in LD to match pairs. Specifically, if one of our significant pairs SNP1-CpG1 could not be directly matched to the replication data, we would search for SNPs in LD (r2 > 0.6) with this SNP1. If we found one SNP in high LD (i.e., SNP2) and SNP2-CpG1 was present in the replication data, then this original pair SNP1-CpG1 could be matched to replication data. In this way, we increased the number of shared pairs among our identified CTS-meQTLs. We also included other methods for comparison (conditional models for bMIND and TCA).

### MeQTL enrichment in genomic functional annotations

For all the variants tested for SNP-CpG associations, we used annotatr [57] and its built-in annotation databases to make CpG annotations (CGI, CGI shelves, CGI shores, inter CGI regions), gene body annotations (regions < 1 kb upstream of the transcription start site, coding sequence, exons, introns, intergenic regions, 5′UTRs, 3′UTRs), gene regulatory and open chromatin annotations (active promotor, weak promotor, strong enhancer, weak enhancer, insulator, regions with heterochromatic or heterochromatin-like characteristics). For gene regulatory and open chromatin annotations [58], we used the database for the K562 cell line, which is commonly used to study hematopoiesis [59]. To test whether the identified meQTLs were enriched in some functional regions, we performed functional enrichment analysis using Fisher’s exact test [60, 61]. A 2 × 2 contingency table was built as follows:

**Table:** 

	**MeQTLs**	**Non-meQTLs**	Row sum
**In functional region R**	MR	R-MR	R
**Not in functional region R**	M-MR	T-R-(M-MR)	T-R
Column sum	M	T-M	T

The total sum of the contingency table (T) was the number of variants that were tested for SNP-CpG associations. The number of identified meQTLs that were mapped in one specific functional region corresponds to the upper-left cell of the table (MR). The remaining three cells of the table can be calculated based on MR and the row/column sums. Based on the 2 × 2 contingency table, we tested whether the meQTLs were enriched in the functional region more often than by chance expected by the genome background (non-meQTLs) [62, 63]. For each cell type, we performed this analysis separately and derived the enrichment estimates as log of odds ratios and its 95% confidence intervals. Enrichment across all cell types was conducted by combining CTS-meQTLs into a union set comprising meQTLs identified in at least one cell type.

### Pathway analyses based on identified CTS-meQTLs

For the identified meQTLs in each cell type, we used ANNOVAR to map variants to their nearest gene, and for variants in intergenic regions, the closest gene was kept [64]. Pathway enrichment analyses were conducted with QIAGEN Ingenuity Pathway Analysis (IPA) (QIAGEN Inc., https://digitalinsights.qiagen.com/IPA) [23]. In each cell type, we reported significant pathways at Bonferroni-adjusted *p* < 0.05.

### Colocalization of meQTL with GWAS loci

To identify potential associations between meQTLs and complex traits, we applied HyPrColoc (Hypothesis Prioritization for multi-trait Colocalization) [26] in multiple genomic regions. We downloaded GWAS summary statistics published by Barbeira et al. [27], and used the 57 traits in the categories of blood cell counts, cardiometabolic, immune, and allergy. Since colocalization reports the posterior probability that two traits are colocalized in a specific linkage disequilibrium (LD) region [26, 65], we first performed clumping on the meQTLs identified in each cell type. For each CpG, highly correlated genetic variants were clustered into one clump with an LD *r*^2^ > 0.1 [66], resulting in 7766 meQTL clumps for CD4+ T-cells, 4211 clumps for CD8+ T-cells, 4568 clumps for monocytes, 3219 clumps for B cells, 2649 clumps for natural killer cells, and 1821 clumps for granulocytes. For each cell type, the genetic variants in each meQTL clump were matched with GWAS summary statistics. Then for each meQTL-GWAS region pair, HyPrColoc was applied on the effect size and the corresponding standard errors. The PPFC was used to identify significant (PPFC > 0.50) colocalizations [35, 67].

### Cell type-specific enrichment in meQTL-GWAS colocalizations

To investigate the cellular specificity of complex traits, we performed enrichment analyses to study if the meQTL-GWAS colocalizations for each trait were enriched in certain cell types. Here, meQTLs in granulocytes were excluded due to low numbers of colocalizations identified across traits, and meQTLs in bulk level (282,965 clumps) were included to assess if CTS-meQTLs can reveal more cellular-specific information. We also excluded three traits with a very small number of meQTL-GWAS colocalizations (< 10) across all cell types. As a result, 54 of the 57 GWAS traits remained in the enrichment analyses. For each trait, in each cell type the enrichment score was defined as the ratio between the percentage of meQTL-GWAS colocalizations (colocalized meQTL clumps) in that cell type and the percentage of meQTL clumps covered by that cell type:

$$Enrichmen{t}_{k}= \frac{\%\,colocalized\,meQTL\,clumps\,in\,cell\,type\,k}{\%\,meQTL\,clumps\,in\,cell\,type\,k}$$

To determine significant colocalization enrichments in certain cell types, the test for equality of proportions with continuity correction was performed to test if $Enrichmen{t}_{k}>1$ (*p* < 0.05/54/6 = 1.5E − 04). To evaluate the identified enrichment results, for the same GWAS traits we also performed heritability enrichment analyses using 66 functional annotations from GenoSkyline-Plus (v1.0.0) [29]. For our identified traits with colocalizations enriched in certain cell types, we determined if the heritability of this trait also enriched in this cell type at *p* < 0.05.

## Supplementary Information

Additional file 1: Supplementary Figures. Supplementary figures 1-10

Additional file 2: Table S1. Demographic information for the WIHS participants

Additional file 3: Table S2. Results for the significant cell-type-specific meQTLs

Additional file 4: Table S3. Replication results for the identified cell-type-specific meQTLs

Additional file 5: Table S4. Canonical Pathways identified using genes mapped by the meQTLs

Additional file 6: Table S5. GWAS-meQTL colocalizations in each cell type

Additional file 7: Table S6. Colocalization enrichment results

Additional file 8: Review history

## Data Availability

Genotype data used in the simulation were downloaded from the Wellcome Trust Case–Control Consortium (https://www.wtccc.org.uk) [68]. Real data used in the simulation were from the ROSMAP gene expression and genomic variants data (https://www.synapse.org/#!Synapse:syn23446022) [21, 69]. Those are third party data. Genotype and DNA methylation data in the application part were from the WIHS cohort, which has been identified as one with multiple vulnerabilities (e.g., racial/ethnic minority women, coinfected). The data was generated by the MWCCS sites (not belong to third party). Whereas participants from the cohort who contributed to the findings summarized in this manuscript provided written consent for genetic studies, said consent was collected prior to the most recent guidelines and requirements for data sharing. The WIHS cohort operates under an alternative data sharing plan registered with the National Institutes of Health and access to data can be requested by submitting a Concept Sheet. The instructions for the Concept Sheet submission could be found at https://www.statepi.jhsph.edu/mwccs/wp-content/uploads/2023/10/MWCCS-Concept-Sheet-and-Publication-Policies_10423.pdf. Investigator(s) should work with the Principal Investigator (PI) of a MWCCS site or MWCCS liaison to draft the concept sheet. External investigators may first request a MWCCS liaison from the Data Analysis and Coordination Center (DACC) at MWCCS@jhu.edu). The accession number for the WIHS in dbGaP genomic data is now provided (phs001503) [70]. The cohort is currently being re-approached to obtain informed consent for sharing of their data. This has been consistent with other genomic studies in the WIHS cohort. The GWAS summary data used in the meQTL-GWAS colocalizations can be downloaded at Zenodo (10.5281/zenodo.3629742) [71]. HBI algorithm is publicly available at https://github.com/YoushuCheng/HBI. The code has also been deposited at Zenodo with 10.5281/zenodo.13131440 [72]. The repository is released under the MIT license.
