# Supplementary material for: HBI: a hierarchical Bayesian interaction model to estimate cell-type-specific methylation quantitative trait loci incorporating priors from cell-sorted bisulfite sequencing data
Source: Genome Biol. 2024 Oct 15;25:273. doi: 10.1186/s13059-024-03411-7 (PMC11476968; doi:10.1186/s13059-024-03411-7)
Supplement: Supplementary file 1 — Additional file 1: Supplementary Figures. Supplementary figures 1-10 [file 13059_2024_3411_MOESM1_ESM.pdf]

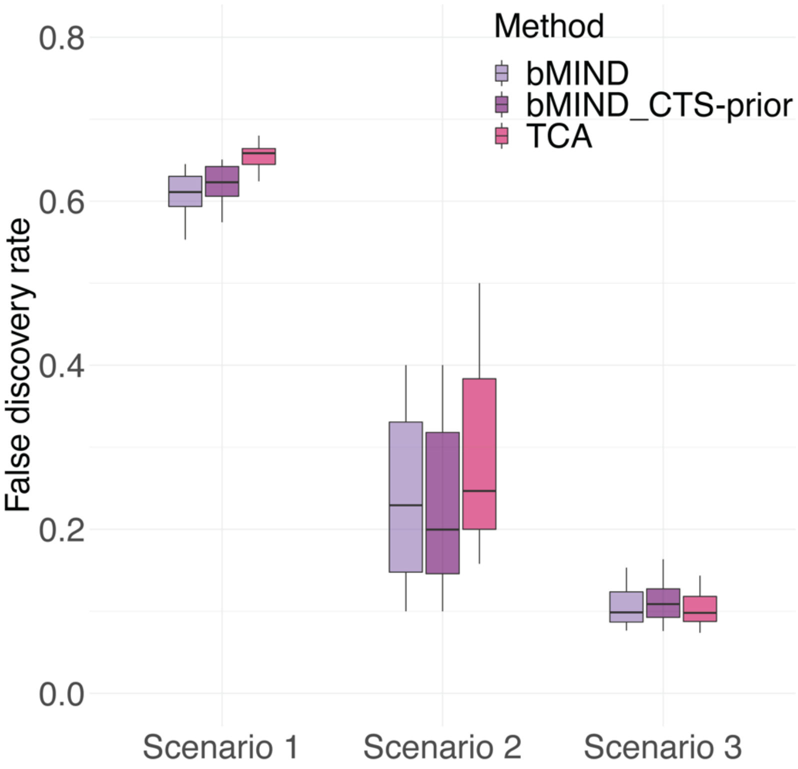


**Fig S1: False discovery rate (FDR) of bMIND and TCA when using the marginal test.** Marginal test refers to the model that regresses the deconvoluted DNAm for one cell type on the genotypes, without controlling for all other cell types. FDR is presented in scenarios with genetic effects only in the most abundant cell type (Scenario 1), only in the least abundant cell type (Scenario 2), and with correlated genetic effects in all cell types (Scenario 3). In each scenario, the proportion of causal SNPs is 20%. bMIND_CTS-prior represents the version of bMIND with cell-type-specific (CTS) methylation data incorporated.

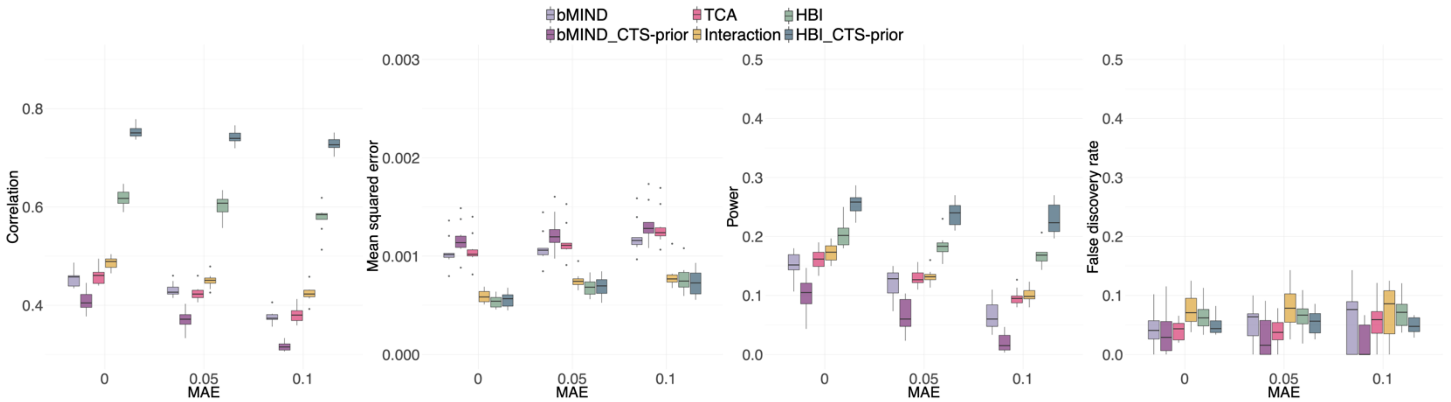


**Fig S2: Performance in estimating cell type specific (CTS)-meQTLs with noisy cell type proportions.** We randomly simulate noise from a normal distribution, add noise to the true cell type proportions, and then normalize the sum of proportions to be 1. We adjust the standard deviation of the added noise so that the generated noisy cell type proportions would have mean absolute error (MAE) of 0.05 and 0.1. From left to right: correlation between estimated and true effect sizes, mean squared error (MSE) between estimated and true effect sizes, power, and false discovery rate as a function of MAE. Scenario 3 (correlated genetic effects in all cell types) is shown, and the proportion of causal SNPs is 20%. HBI_CTS-prior, bMIND_CTS-prior represent the version of the corresponding methods with CTS methylation data incorporated.

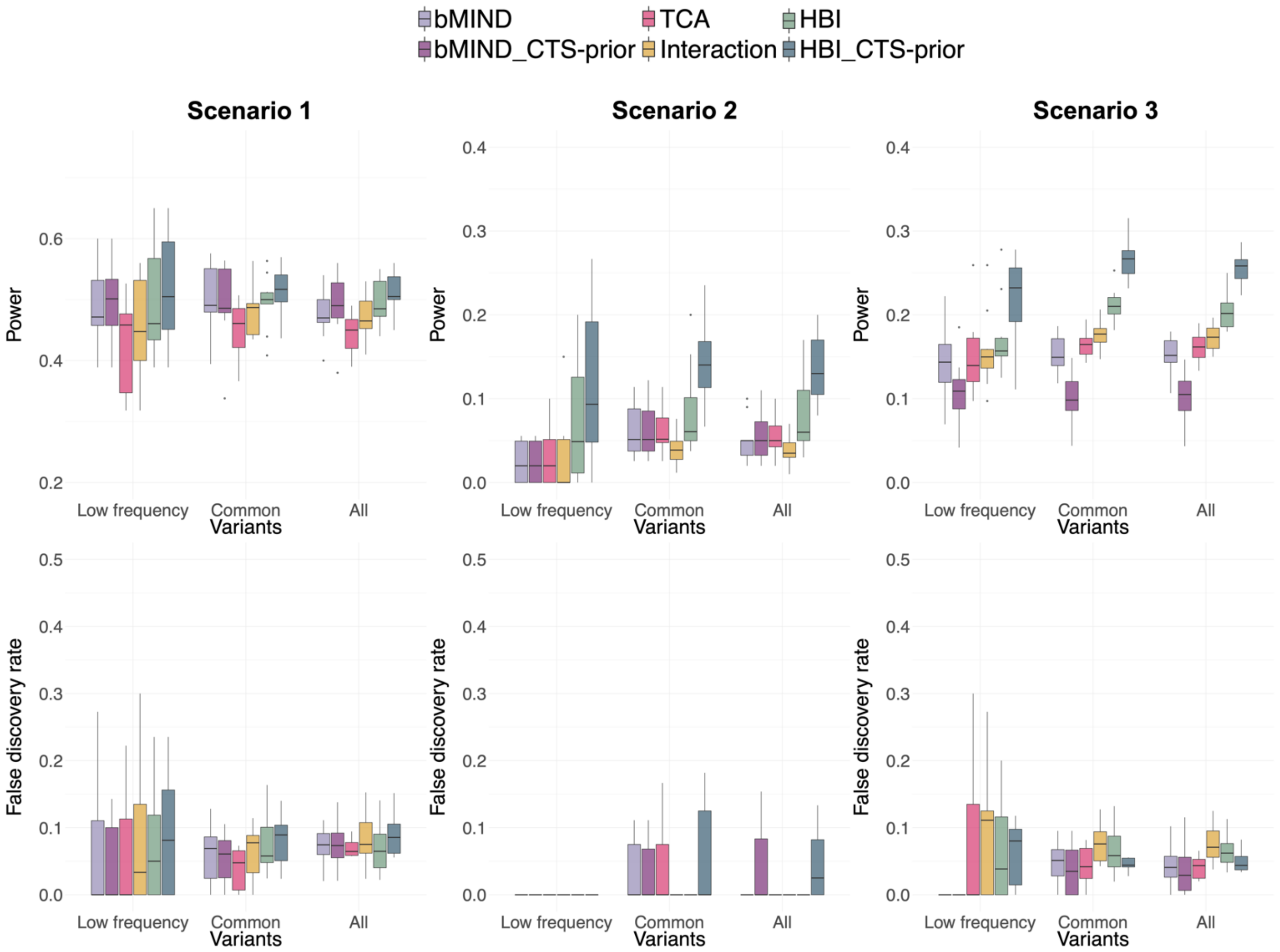


**Fig S3: Performance in estimating cell type specific (CTS)-meQTLs with different minor allele frequencies (MAF) for the variants.** From top to bottom: power, and false discovery rate (FDR) versus variants in different groups: low frequency variants (0.01≤MAF<0.05), common variants (MAF≥0.05), and all variants combining the two groups. From left to right: scenarios with genetic effects only in the most abundant cell type (Scenario 1), only in the least abundant cell type (Scenario 2), and with correlated genetic effects in all cell types (Scenario 3) are shown. The proportion of causal SNPs is 20%. HBI_CTS-prior, bMIND_CTS-prior represent the version of the corresponding methods with CTS DNA methylation data incorporated.

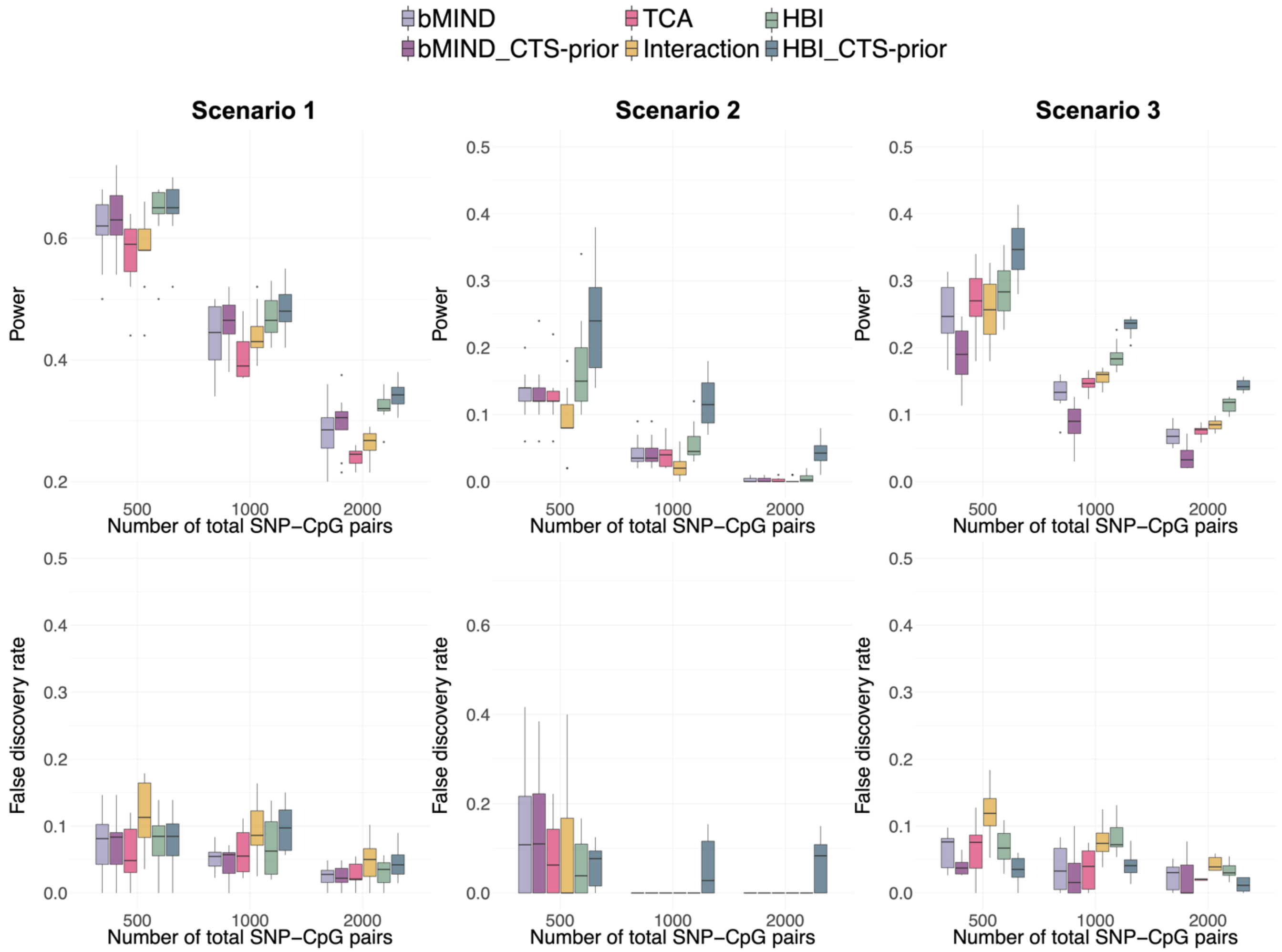


**Fig S4: Performance in estimating cell type specific (CTS)-meQTLs with different numbers of total SNPs near one CpG.** From top to bottom: power, and false discovery rate (FDR) as a function of the number of total SNP-CpG pairs. From left to right: scenarios with genetic effects only in the most abundant cell type (Scenario 1), only in the least abundant cell type (Scenario 2), and with correlated genetic effects in all cell types (Scenario 3) are shown. The proportion of causal SNPs is 10%. HBI_CTS-prior, bMIND_CTS-prior represent the version of the corresponding methods with CTS DNA methylation data incorporated.

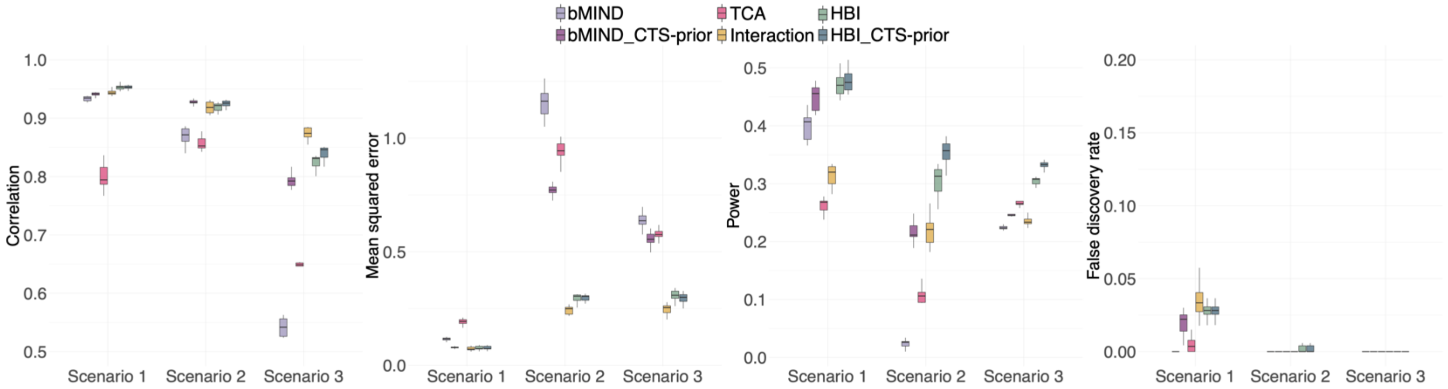


**Fig S5: Performance in estimating cell type specific (CTS)-eQTLs using the ROSMAP study data.** The ground truth of the CTS eQTLs was first estimated using the single cell RNA seq data, and then we created pseudo-bulk data as the input for all the methods. From left to right: correlation between estimated and true effect sizes, mean squared error (MSE) between estimated and true effect sizes, power, and false discovery rate in different scenarios (Scenario 1: genetic effects only in the most abundant cell type, Scenario 2: genetic effects only in the least abundant cell type, Scenario 3: correlated genetic effects in all cell types). HBI_CTS-prior, bMIND_CTS-prior represent the version of the corresponding methods with CTS gene expression data incorporated.

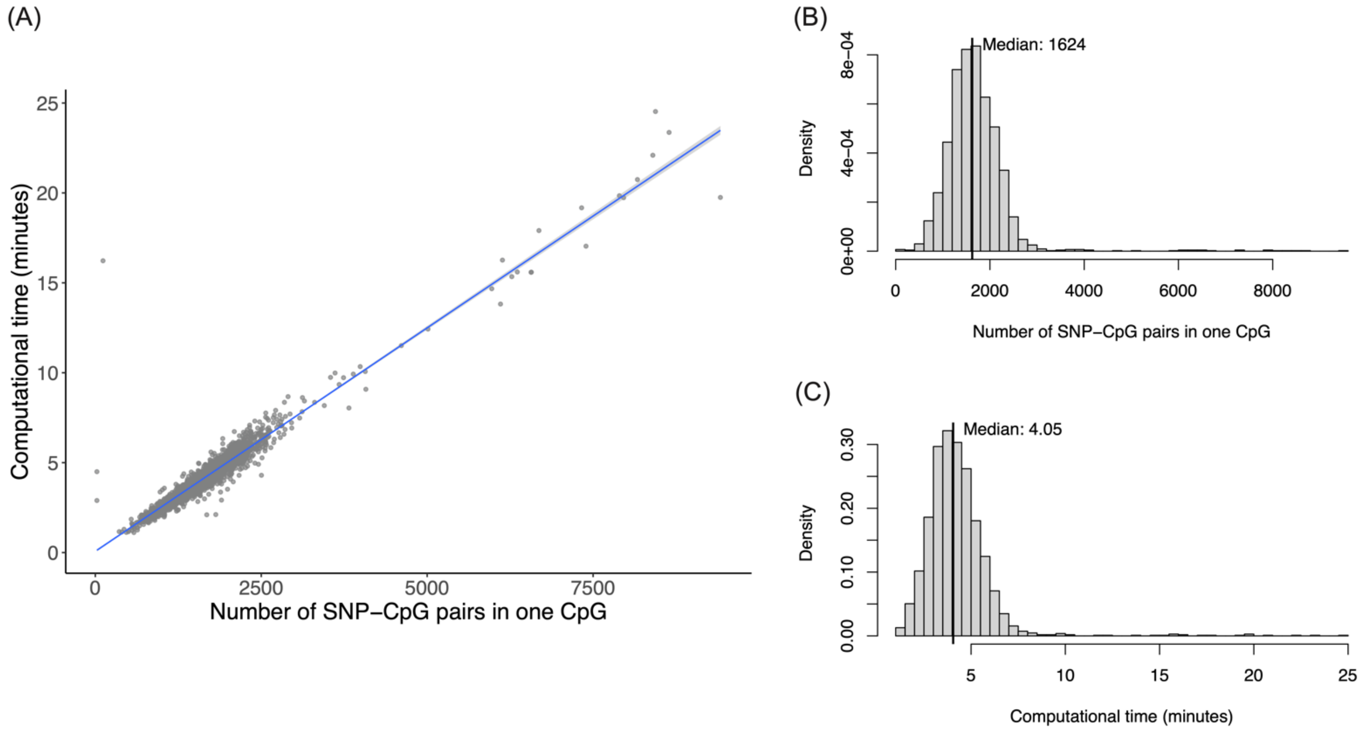


**Fig S6: Computational time of HBI. (A)** The line was fitted by regressing the computational time (in minutes) on the number of SNP-CpG pairs in one CpG. HBI was applied on the Women's Interagency HIV Study (WIHS) (n=431). **(B)** The histogram shows the distribution of the number of SNP-CpG pairs for one CpG, and the median is 1624. (**C**) The histogram shows the distribution of the computational time for one CpG, and the median is 4.05 minutes.

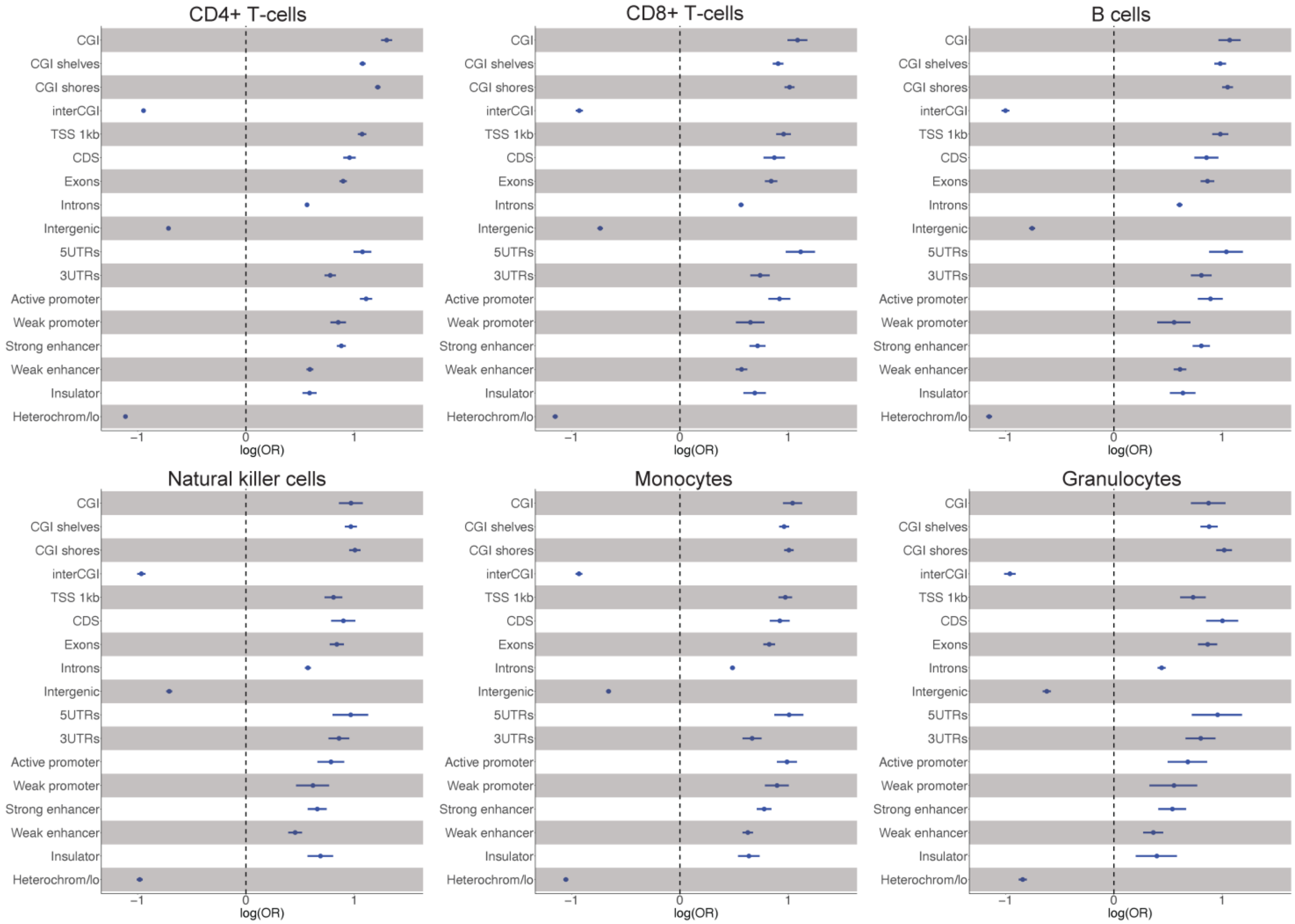


**Fig S7: Functional enrichment for cell type specific (CTS)-meQTLs in CpG island (CGI) regions, gene body regions, and gene regulatory regions.** The logarithm of odds ratio (OR) with 95% confidence interval is presented. TSS 1kb: <1kb upstream of the transcription start site (TSS); CDS: coding sequence; UTR: untranslated exon region; Heterochrom/lo: regions that exhibit heterochromatic or heterochromatin-like characteristics.

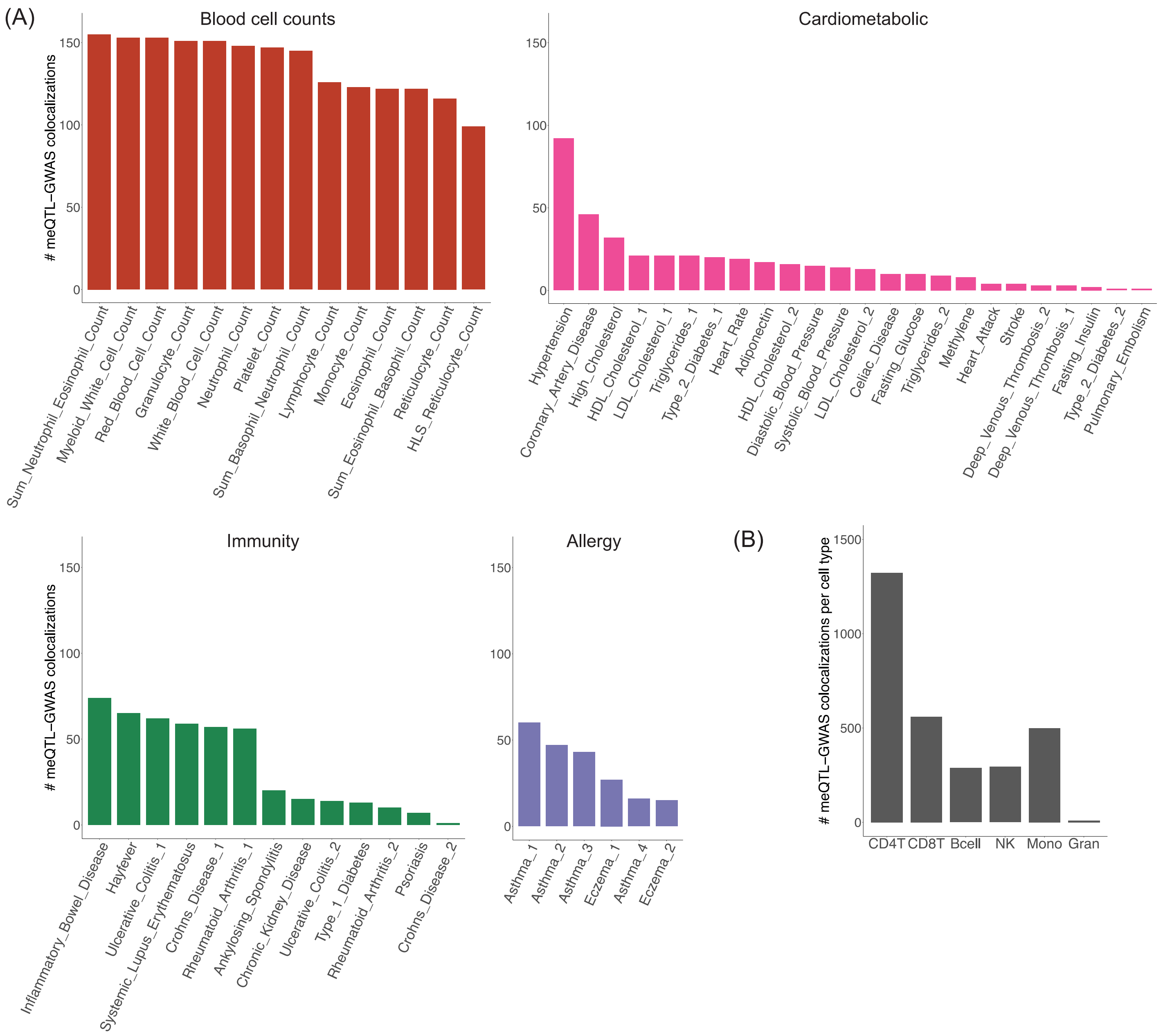


**Fig S8: Colocalization of meQTLs with GWAS traits. (A)** Bar plot represents the number of meQTL-GWAS colocalizations per GWAS trait across all six cell types. **(B)** Bar plot represents the number of meQTL-GWAS colocalizations per cell type across all GWAS traits.

HLS reticulocyte: high light scatter reticulocyte; HDL: high-density lipoprotein; LDL: low-density lipoprotein; GWAS: genome-wide association studies; CD4T: CD4+ T-cells; CD8T: CD8+ T-cells; NK: natural killer cells; Mono: monocytes; Gran: granulocytes.

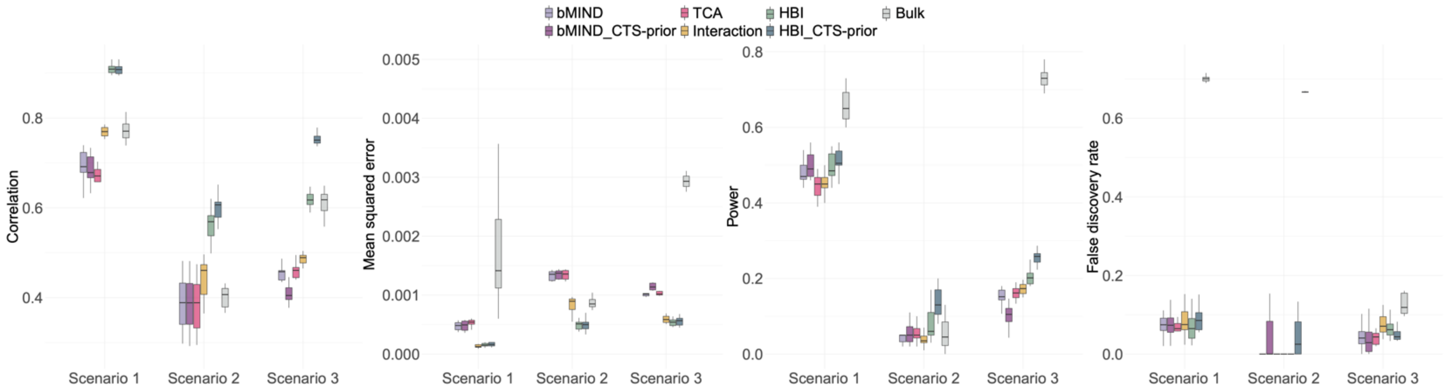


**Fig S9: Comparison between cell type specific (CTS)-meQTLs and bulk meQTLs identified using the bulk data.** Bulk meQTLs are identified using the linear regression model that directly regresses bulk methylation on genotypes. To compare with CTS-meQTLs, we assume the identified bulk meQTLs are the same (homogeneous) in all cell types. Correlation, mean squared error (MSE) between estimated and true effect sizes, power, and false discovery rate are presented in scenarios with genetic effects only in the most abundant cell type (Scenario 1), only in the least abundant cell type (Scenario 2), and with correlated genetic effects in all cell types (Scenario 3). In each scenario, the proportion of causal SNPs is 20%. HBI_CTS-prior, bMIND_CTS-prior represent the version of the corresponding methods with CTS methylation data incorporated.

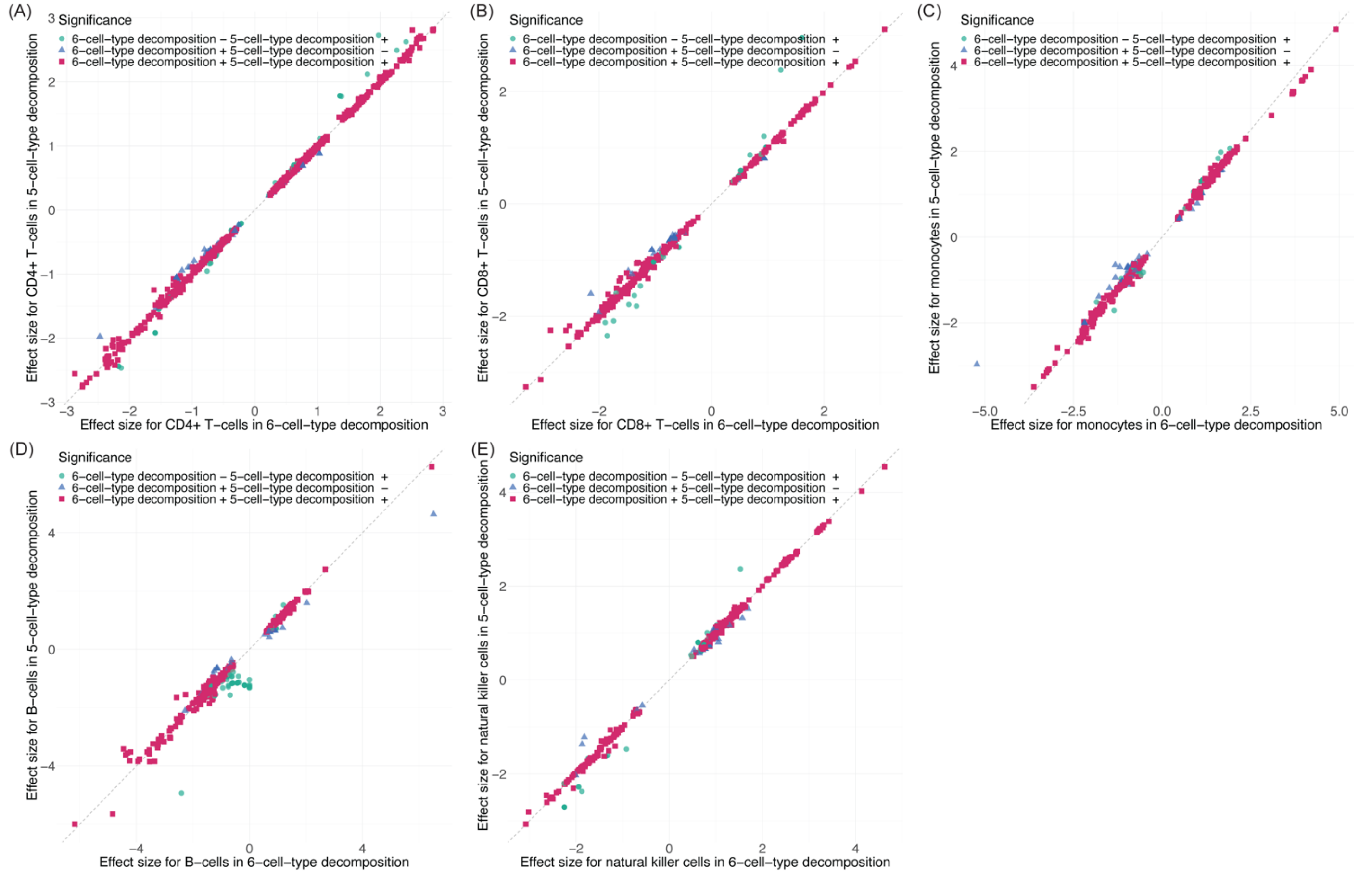


**Fig S10: Comparison of meQTL effect sizes between 6-cell-type decomposition and 5-cell-type decomposition (without granulocytes).** A sensitivity analysis was conducted with 5-cell-type decomposition (proportion of granulocytes removed). The effect sizes in CD4+ T-cells, CD8+ T-cells, monocytes, B-cells, and natural killer cells were compared. MeQTLs that are significant in either 6-cell-type decomposition or 5-cell-type decomposition, or both are plotted. The correlations of effect size were 0.99, 0.99, 0.99, 0.98, 0.99 for CD4+ T-cells, CD8+ T-cells, monocytes, B-cells, and natural killer cells, respectively.
