# Supplementary material for: HBI: a hierarchical Bayesian interaction model to estimate cell-type-specific methylation quantitative trait loci incorporating priors from cell-sorted bisulfite sequencing data
Source: Genome Biol. 2024 Oct 15;25:273. doi: 10.1186/s13059-024-03411-7 (PMC11476968; doi:10.1186/s13059-024-03411-7)
Supplement: Supplementary file 8 — Additional file 8: Review history [file 13059_2024_3411_MOESM8_ESM.docx]

**1^st^ round**

**Reviewer 1**

In this manuscript, the authors propose a hierarchical Bayesian interaction (HBI) model to identify methylation quantitative trait loci (me-QTL). The proposed method differs from existing ones by modeling cell-specific effects of potential me-QTL via gene-by-cell type interactions. It also leverages Bayesian framework to integrate bulk methylation data and cell-sorted bisulfite sequencing data when available. Through simulation, the authors demonstrated that the proposed method had improved performance. The application study also illustrated the method can improve the identification of cell-type specific meQTLs and their functional annotation. I believe the proposed method can be a timely addition to the existing tools for integrative genomic analyses and is likely to be widely used in many studies. I only have a few minor/moderate comments:

1. Simulation settings. What are the total numbers of CpGs? Each simulation setting was repeated 10 times. Does it mean 500 CpG-SNP pair in each repeat, which seems to be a very small number. How were 500 variants being selected and what are their allele frequencies? It seems to me that variants with lower allele frequencies are most likely to be associated with low power and high false positives. Can you provide some results to evaluate the performance related to allele frequencies?

2. The real data application included ~2.4 million variants and ~850K CpG sites, and ~1.4 billion SNP-CpG pairs were tested. Were these tests to detect cis-QTL, trans-QTL or both? What is the criteria for a pair to be tested?

3. It seems counter-intuitive that the identified CTS meQTLs colocalize with rare variants since all the rare variants (MAF>0.05) have been removed from the analysis. One example was given in the result, but it is not clear how this conclusion is reached.

4. Some discussion on the computation time and description of software availability would be needed.

**Reviewer 2**

Cheng et al presents their work on the development and implementation of a new hierarchical Bayesian interaction (HBI) model to estimate cell-type-specific methylation quantitative trait loci. While several methods have been established for this purpose the authors show convincing data that the HBI model outperforms many of these. However, a major limitation with this method (and others) is the requirement of cell proportions which mostly is available for blood samples which limits the use of the approach. In addition, while blood-based cohort is by far the most commonly used tissue type for mQTL studies, cell proportions are not always available. There are several other concerns outlined below that further dampens the enthusiasm for the paper.

1. The statistical power of HBI is sufficient only for certain scenarios including 1) when genetic association is seen for only the most abundant cell type and 2) when only a limited number of SNPs control DNA methylation variation in that cell type. While simulations can use these assumptions, biological scenarios are more complex than these computational models and should be outlined. To be specific, how many CpGs identified would fit in these two scenarios and thus HBi would be able to resolve associations? The authors should use real data available in repositories for their model developments and not arbitrary chose assumptions and scenarios without biological explanations.
2. The authors indicate that their HBI method applied to PBMCs identified ~12K meQTLs specific to granulocytes. Of note is that the PBMC isolation method per se filters out granulocytes and should only include monocytes, B and T cells so the authors need to explain these findings. Does this indicate additional noise in their method?
3. Given the limitation with having cell subset in large blood based mQTL studies, the author should report the replication effort of their HBI method using the WIHS cohort without the 47 samples having CTS separation for comparisons.
4. The number of shared pairs in replication attempt is very low (~13% across the cell types) which appears puzzling and needs to be explained in further detail, especially as the methylation detection approach is the same (EPIC Array)?! SNPs in high LD should be utilized to increase the pairs. In addition, other methods should be included here for comparisons (i.e. TCA, bMIND).
5. There are larger mQTLs efforts published in blood cell types which the authors need to utilize for replication, i.e. PMID: 27863251
6. The GWAS overlap with T1D example needs some more clarifications - SNPs in the HLA region is the most important inherited risk factors for T1D and with no other GWAS studies than the one used by the authors (UKBB) showing association with the particular SNP may indicate this is driven by the major haplotypes.

**Authors’ response**

Reviewer #1:

In this manuscript, the authors propose a hierarchical Bayesian interaction (HBI) model to identify methylation quantitative trait loci (me-QTL). The proposed method differs from existing ones by modeling cell-specific effects of potential me-QTL via gene-by-cell type interactions. It also leverages Bayesian framework to integrate bulk methylation data and cell-sorted bisulfite sequencing data when available. Through simulation, the authors demonstrated that the proposed method had improved performance. The application study also illustrated the method can improve the identification of cell-type specific meQTLs and their functional annotation. I believe the proposed method can be a timely addition to the existing tools for integrative genomic analyses and is likely to be widely used in many studies. I only have a few minor/moderate comments:

***Response:*** *We appreciate the reviewer’s encouraging comments.*

1.      Simulation settings. What are the total numbers of CpGs? Each simulation setting was repeated 10 times. Does it mean 500 CpG-SNP pair in each repeat, which seems to be a very small number. How were 500 variants being selected and what are their allele frequencies? It seems to me that variants with lower allele frequencies are most likely to be associated with low power and high false positives. Can you provide some results to evaluate the performance related to allele frequencies?

***Response****: Thank you for your questions and suggestion. Yes, in each repeat we simulated one CpG as the phenotype and assumed that the total number of SNPs near the simulated CpG site was 500 (so 500 SNP-CpG pairs in each repeat). Those 500 variants were randomly selected from chromosome 12 and all have minor allele frequency (MAF) > 0.01 (rare variant with MAF < 0.01 were removed in QC). We agreed that the MAF of variants can affect power and false discovery rate, and it is also important to assess the performance with a larger number of SNP-CpG pairs in each repeat. Therefore, we further extended the simulations to investigate the performance under (a) different allele frequencies, and (b) different number of SNP-CpG pairs.*

*(a) To investigate the effect of variants with lower frequency, we divided all SNPs into variants with low frequency (0.01≤MAF<0.05) and common variants (MAF≥0.05) and assessed their performance separately. From* ***Supplementary Figure 3****, we found that variants with low frequency are more likely to have lower power, especially in scenario 2 (genetic effects only in the least abundant cell type). In scenario 1 (genetic effects only in the most abundant cell type) and scenario 3 (correlated genetic effects in all cell types), variants with low frequency are more likely to have inflated false discovery rate and slightly lower power compared to common variants.*

*(b) To assess the effect of the number of SNPs near the simulated CpG site (SNP-CpG pairs), we increased the number of pairs from 500, 1000, to 2000. We observed that power decreased with an increasing number of SNP-CpG pairs (****Supplementary Figure 4****). This is expected: with heritability (proportion of methylation variation that is explained by SNPs) fixed and the number of SNPs increased, the genetic effects change from sparse to polygenic (diluted on a larger number of SNPs), and it became harder to detect signals.*

*HBI generally had better performance with improved power across all these settings.*

*Changes were made in the manuscript lines 223-225, 592-596, 639-642, and* ***Supplementary Figure 3-4*** *was added.*

**Supplementary Figure 3: Performance in estimating cell type specific (CTS)-meQTLs with different minor allele frequencies (MAF) for the variants.** From top to bottom: power, and false discovery rate (FDR) versus variants in different groups: low frequency variants (0.01≤MAF<0.05), common variants (MAF≥0.05), and all variants combining the two groups. From left to right: scenarios with genetic effects only in the most abundant cell type (Scenario 1), only in the least abundant cell type (Scenario 2), and with correlated genetic effects in all cell types (Scenario 3) are shown. HBI_CTS-prior, bMIND_CTS-prior represent the version of the corresponding methods with CTS DNA methylation data incorporated.

**Supplementary Figure 4: Performance in estimating cell type specific (CTS)-meQTLs with different numbers of total SNPs near one CpG.** From top to bottom: power, and false discovery rate (FDR) as a function of the number of total SNP-CpG pairs. From left to right: scenarios with genetic effects only in the most abundant cell type (Scenario 1), only in the least abundant cell type (Scenario 2), and with correlated genetic effects in all cell types (Scenario 3) are shown. The proportion of causal SNPs is set to 10%. HBI_CTS-prior, bMIND_CTS-prior represent the version of the corresponding methods with CTS DNA methylation data incorporated.

2.      The real data application included ~2.4 million variants and ~850K CpG sites, and ~1.4 billion SNP-CpG pairs were tested. Were these tests to detect cis-QTL, trans-QTL or both? What is the criteria for a pair to be tested?

***Response****: Thank you for your questions. For each CpG site, we tried to identify its cis-meQTLs, with SNPs within 500kb upstream and 500kb downstream of the site included. This window size has been widely used to identify cis-meQTLs [1-4]. We added more references in the manuscript to clarify this.*

*Changes were made in the manuscript lines 694-695.*

3.      It seems counter-intuitive that the identified CTS meQTLs colocalize with rare variants since all the rare variants (MAF>0.05) have been removed from the analysis. One example was given in the result, but it is not clear how this conclusion is reached.

***Response****: Sorry for the confusion, we did not intend to conclude that “CTS meQTLs colocalize with* ***rare*** *variants”. Instead, we investigated whether the identified CTS meQTLs colocalized with (common)* ***risk*** *variants for complex traits (as the subtitle in line 285). We have removed rare variants (MAF<0.05) prior to the genome-wide CTS-meQTLs identification.*

*We further checked the colocalized SNP in the given example (rs2395178,* ***Figure 4****), the MAF was 0.39 in our meQTL cohort. In the GWAS summary statistics, the MAF of rs2395178 was reported to be 0.33 [5]. This further demonstrated that no rare variants were included.*

*We made it more clear in the manuscript line 285.*

4.      Some discussion on the computation time and description of software availability would be needed.

***Response****: Thank you for your suggestion. The computation time was added to the Results and Discussion session. Specifically, the computational time increased linearly with the number of SNP-CpG pairs for a specific CpG (****Supplementary Figure 6A****). The median number of pairs for one CpG was 1624 while the median computational time was 4.05 minutes (****Supplementary Figure 6B, C****).*

*The software availability was added to the Availability of data and materials session under Declarations.*

*Changes were made in the manuscript lines 245-246, 440-443, 822-824, and* ***Supplementary Figure 6*** *was added.*

**Supplementary Figure 6: Computational time of HBI. (A)** The line was fitted by regressing the computational time (in minutes) on the number of SNP-CpG pairs in one CpG. HBI was applied on the Women’s Interagency HIV Study (WIHS). **(B)** The histogram shows the distribution of the number of SNP-CpG pairs for one CpG, and the median is 1624. (**C**) The histogram shows the distribution of the computational time for one CpG, and the median is 4.05 minutes.

Reviewer #2:

Cheng et al presents their work on the development and implementation of a new hierarchical Bayesian interaction (HBI) model to estimate cell-type-specific methylation quantitative trait loci. While several methods have been established for this purpose the authors show convincing data that the HBI model outperforms many of these. However, a major limitation with this method (and others) is the requirement of cell proportions which mostly is available for blood samples which limits the use of the approach. In addition, while blood-based cohort is by far the most commonly used tissue type for mQTL studies, cell proportions are not always available. There are several other concerns outlined below that further dampens the enthusiasm for the paper. 

***Response:*** *We appreciate the reviewer’s insightful comments. We agreed with the limitation mentioned by the reviewer: the cell type proportions were not always available. To apply HBI (and other methods), we may need to estimate the cell type proportions in the first place. For now, the method described by Houseman et al. [6, 7] was widely applied to estimate cell proportions for DNA methylation studies in blood samples [8, 9]. There are also some efforts to quantify the cell proportion for non-blood samples (i.e., brain samples) [10]. Therefore, with those algorithms to estimate cell proportions, HBI and other methods can then be applied, which allow them to be used on a relatively wide range of datasets. However, those proportions were not truly measured and relying on them may introduce inaccuracy. More details on this were discussed in the response to comment 2. Additional analyses were conducted, and other concerns were discussed in the following point-by-point responses.*

1.      The statistical power of HBI is sufficient only for certain scenarios including 1) when genetic association is seen for only the most abundant cell type and 2) when only a limited number of SNPs control DNA methylation variation in that cell type. While simulations can use these assumptions, biological scenarios are more complex than these computational models and should be outlined. To be specific, how many CpGs identified would fit in these two scenarios and thus HBi would be able to resolve associations? The authors should use real data available in repositories for their model developments and not arbitrary chose assumptions and scenarios without biological explanations.

***Response****: Thank you for your insightful comments and suggestions. We agree that utilizing some real data in the simulations is very helpful to further investigate and compare different methods. Our original simulation settings were built mainly to compare the power of HBI and other state-of-the-art methods. For example, we varied the proportion of causal SNPs from 10% to 40%. In reality, QTL signals tend to be sparse [11-14]. In the cosmopolitan meQTL results reported by Hawe et al. [15], the median number of significant meQTLs for one CpG was 72. Assuming the total number of SNPs for one CpG within 500kb upstream and 500kb downstream was 1624 (****Supplementary Figure 6B****), the median proportion of causal SNPs for one CpG would be 4.4% (72/1624), and approximately 71.9% CpGs would have the proportion of causal SNPs below 10% (fit in the scenario that a limited number of SNPs control DNA methylation variation). Although the proportion of causal SNPs will rarely be as high as 40%, we kept this polygenic setting (the effects were diluted to a large number of SNPs and the detection of associations is more challenging) to see whether HBI can still have higher power over other methods in this “difficult” setting. Therefore, we focused more on the comparison of power rather than the absolute power of HBI.*

*As the reviewer rightfully pointed out, the biological scenarios are complex than our assumptions, and relying only on the simulations or assumptions might be insufficient. To fill this gap, we searched for potential publicly available real data, which requires matched genotype and CTS DNA methylation data for the same individuals. In this way, we can first use the CTS DNA methylation data to estimate “ground truth” and then combine the CTS profile as pseudo-bulk to test the performance of HBI and other methods. Thus, the results can be compared to the estimated “ground truth”. However, few public data sets on CTS DNA methylations are available in large sample sizes. We also noted that by principle, HBI can also be used to identify CTS expression quantitative trait loci (eQTL). To address the reviewer’s concern, we finally used the ROSMAP gene expression and genotype data [16] (with CTS gene expression data from its single cell RNA seq data) (n=290). The ROSMAP data was used as alternative to validate the HBI performance in the real dataset.*

*We first estimated the ground truth (CTS-eQTLs) using the single cell RNA seq data. We then extracted the significant eQTLs (Bonferroni-adjusted p<0.05) fitting into three scenarios: (1) eQTLs only in excitatory neurons (simulated as the major cell type in pseudo-bulk), (2) eQTLs only in oligodendrocytes (simulated as the minor cell type in pseudo-bulk), and (3) eQTLs in all cell types. The effect sizes of those eQTLs estimated by single cell RNA seq data were treated as “ground truth”. Pseudo-bulk data was then created as the input for TCA, bMIND, the basic interaction model and HBI. Similarly, to evaluate the power of those methods to correctly identify those eQTLs in each scenario, we calculated the correlation between the estimated and true effect sizes, the MSE between the estimated and true effect sizes, power, and FDR. The results were summarized in* ***Supplementary Figure 5*** *(attached below), with* *similar patterns observed: HBI generally achieved higher correlation and lower MSE, and also had higher power across different scenarios.*

*We also note that in this* *real data simulation part, the ground truth of CTS-eQTLs was estimated using the single cell RNA seq data. This may introduce some inaccuracy, especially for the gene-SNP pairs that were not significant in the single cell RNA seq data. They could be true nulls, or false negatives due to the limited power, and we were not able to differentiate the 2 conditions as the exact ground truth was unknown. Therefore, we focused on the significant eQTLs (Bonferroni-adjusted p<0.05) identified using the single cell RNA seq data, and investigated how many of them could be correctly identified by HBI or other methods. In contrast, the exact ground truth was simulated and known in our original simulations, in which case we did not have the inaccuracy introduced by the estimated “ground truth”. Taken together, the original simulations and the added real data simulation can be complementary to each other and provide a comprehensive evaluation of HBI and other methods.*

*More details were added to the* ***Methods*** *and* ***Results*** *session in the manuscript lines 226-232, 643-657, 841-853 and* ***Supplementary Figure 5*** *was added.*

**Supplementary Figure 5: Performance in estimating cell type specific (CTS)-eQTLs using the ROSMAP study data.** The ground truth of the CTS eQTLs was first estimated using the single cell RNA seq data, and then we created pseudo-bulk data as the input for all the methods. From left to right: correlation between estimated and true effect sizes, mean squared error (MSE) between estimated and true effect sizes, power, and false discovery rate in different scenarios (Scenario 1: genetic effects only in the most abundant cell type, Scenario 2: genetic effects only in the least abundant cell type, Scenario 3: correlated genetic effects in all cell types). HBI_CTS-prior, bMIND_CTS-prior represent the version of the corresponding methods with CTS gene expression data incorporated.

2.      The authors indicate that their HBI method applied to PBMCs identified ~12K meQTLs specific to granulocytes. Of note is that the PBMC isolation method per se filters out granulocytes and should only include monocytes, B and T cells so the authors need to explain these findings. Does this indicate additional noise in their method?

***Response****: Thank you for your insightful comment. In our data, the granulocytes were indeed rare: the average proportion was 0.01 (sd=0.04) (****Supplementary Table 1****), indicating there was low amount of granulocytes in the estimated cell type proportions. The potential reasons could be: (1) there is slight granulocyte contamination during PBMC processing [17], so low amount of granulocytes exist in our samples; (2) the cell type proportion was estimated by the method described by Houseman et al. [6, 7] and was not “truly” measured value. The algorithm had some inaccuracies, which overestimated the cell type proportions of granulocytes. Considering that the proportions estimated as “granulocytes” might also represent the unclassified proportions that cannot be classified into other main cell types, we kept the granulocytes together with other 5 cell types in our model (6-cell-type decomposition), while the results identified for granulocytes were not our focus. We agree that it is also helpful to repeat the analyses without granulocytes in the model (5-cell-type decomposition) and investigate the impacts on the results. Therefore, we conducted a sensitivity analysis with 5-cell-type decomposition. Highly consistent results were observed, with the correlations of effect size above 0.98 in the 5 main cell types (****Supplementary Figure 10****).*

*Therefore, the identified meQTLs in granulocytes (which should be filtered in PBMC theoretically) can be a result of technical issues (granulocyte contamination during PBMC processing, or the inaccuracy in the estimated cell type proportions), and including or excluding this cell type in the model did not have a big impact on the results.*

*We also agreed that truly measured cell proportions were not always available. To apply HBI (and other methods), we need to estimate the cell type proportions in the first place. For now, the method described by Houseman et al. [6, 7] was widely applied to estimate cell proportions for DNA methylation studies in blood samples [8, 9]. There are also some efforts to quantify the cell proportion for non-blood samples (i.e., brain samples) [10]. Therefore, with those algorithms to estimate cell proportions, HBI and other methods can then be applied, which allow them to be used on a relatively wide range of datasets. However, those estimated proportions would be directly used as the biological ground truth, which may introduce additional error (i.e., may overestimate the proportions of granulocytes here). To solve this technical issue, one future direction is to incorporate the uncertainty for the estimated cell type proportion. Specifically, we will extend the statistical model to estimate/refine the cell type proportions and adjust for the uncertainty in the estimated proportions at the same time. We will focus on this direction in the near future. This would help us to have wider applications to non-blood samples as we will not rely on other algorithms to estimate cell proportions, and obtain more accurate results as the uncertainty in the estimated proportions is considered and adjusted.*

*More discussion and explanations were added to the manuscript.*

*Changes were made in the manuscript lines 445-457, 709-712, and* ***Supplementary Figure 10*** *was added.*

**Supplementary Figure 10: Comparison of meQTL effect sizes between 6-cell-type decomposition and 5-cell-type decomposition (without granulocytes).** A sensitivity analysis was conducted with 5-cell-type decomposition (proportion of granulocytes removed). The effect sizes in CD4+ T-cells, CD8+ T-cells, monocytes, B-cells, and natural killer cells were compared. MeQTLs that are significant in either 6-cell-type decomposition or 5-cell-type decomposition, or both are plotted. The correlations of effect size were 0.99, 0.99, 0.99, 0.98, 0.99 for CD4+ T-cells, CD8+ T-cells, monocytes, B-cells, and natural killer cells, respectively.

3.      Given the limitation with having cell subset in large blood based mQTL studies, the author should report the replication effort of their HBI method using the WIHS cohort without the 47 samples having CTS separation for comparisons.

***Response****: Thank you for your advice. We have added the replication results using only the bulk data without priors incorporated from CTS data. The results were updated in* ***Supplementary Table 3A*** *(also attached below). In general, the replication rates did not change much if there was no CTS information. The advantage of incorporating CTS data mainly reflected on the increased number of significant meQTLs. For example, with priors incorporated from CTS data, HBI increased the number of significant meQTLs from 31,086 to 34,310 (10.4%) in CD8+ T-cells and from 34,016 to 36,919 (8.5%) in monocytes, while the improvement in CD4+ T-cells (the most abundant cell type) was relatively small. This was also consistent with the simulation results: HBI with CTS DNAm data incorporated can help to improve the power, especially in less abundant cell types.*

*Changes were made in the revised manuscript lines 258-259, 723-725, and* ***Supplementary Table 3A*** *was added.*

**Supplementary Table 3A:  Replication results for HBI with and without priors incorporated from CTS data.** *Replicated meQTLs were defined as those with p<0.05 and consistent direction of effect in the replication data. Replication rate was calculated as (number of replicated meQTLs)/(number of shared pairs).

4.      The number of shared pairs in replication attempt is very low (~13% across the cell types) which appears puzzling and needs to be explained in further detail, especially as the methylation detection approach is the same (EPIC Array)?! SNPs in high LD should be utilized to increase the pairs.  In addition, other methods should be included here for comparisons (i.e. TCA, bMIND).

***Response****: Thank you for pointing this out. There are two main reasons for the low number of shared pairs: (1) CpG data in the replication set was generated using the Illumina Human Methylation 450K BeadChip [15] while our results were based on the Illumina Infinium Methylation EPIC BeadChip. (2) The CTS-meQTLs results released by Hawe et al. [15] comprised of 11.2 million SNP-CpG pairs, which was significant results in their previous meQTL analyses in peripheral blood (bulk). Therefore, the released data was based on a subset of the genome-wide SNP-CpG pairs that was preselected in the authors’ previous analyses. As a result, not all our significant results were represented in their database. We acknowledged that this was a limitation and added more explanation in the Discussion session.*

*We agreed that utilizing high LD SNPs could increase the number of shared pairs, and we conducted parallel analyses after utilizing high LD SNPs. The results were summarized in* ***Supplementary Table 3B*** *(also attached below). For HBI, the inclusion of high LD SNPs increased the number of shared pairs from 16,088 to 23,694 in CD4+ T-cells, from 3,159 to 4,222 in CD8+ T-cells, and from 5,430 to 6,804 in monocytes. Compared with the results using the exactly matched pairs, the replication rates using high LD SNPs showed a slight decrease in CD8+ T-cells and monocytes (****Supplementary Table 3B****), partially due to the noise introduced by those SNPs in LD.*

*We also included results from TCA and bMIND. In general, the replication rates were comparable: the three methods had similar replication rates for CD4+ T-cells, while bMIND had the highest rate for CD8+ T-cells and HBI had the highest rate for monocytes (****Supplementary Table 3B****). The advantage of HBI mainly reflected on the increased number of significant meQTLs. For example, in CD4+ T-cells, the number of significant meQTLs (122,387) in HBI was similar to bMIND (120,846), and higher than TCA (77,084). In CD8+ T-cells and monocytes, HBI identified more significant meQTLs than bMIND and TCA (34,310 and 36,919 for HBI, 30,214 and 21,922 for bMIND, 25,152 and 28,408 for monocytes, respectively). To sum up, HBI helped to identify more significant results especially in less abundant cell types (consistent with simulations) while keeping the replication rates comparable.*

*Changes were made in the manuscript lines 259-261, 459-464, 725-732, and* ***Supplementary Table 3B*** *was added.*

**Supplementary Table 3B:** **Replication results for different methods before and after utilizing high LD SNPs to increase the number of shared pairs.**

5.      There are larger mQTLs efforts published in blood cell types which the authors need to utilize for replication, i.e. PMID: 27863251

***Response****: Thank you for the advice and the suggested reference data. We added this additional data with larger sample sizes (n=197 individuals) for meQTLs in isolated blood cells (CD4+ T-cells, monocytes) in our replication [18]. Of note, this data was generated using the Illumina Human Methylation 450K BeadChip, and we also used the same way to include SNPs in high LD. The results were summarized in* ***Supplementary Table 3C*** *(attached below).*

*Compared with the replication results using the original data, the new results generally showed higher replication rate. For example, the replication rates for HBI were 93.93% in CD4+ T-cells and 88.05% in monocytes using the original replication set (****Supplementary Table 3B****), and increased to 97.31% and 92.40% using the new replication set (****Supplementary Table 3C****). The other patterns were consistent: utilizing high LD SNPs increased the number of shared pairs; HBI identified more significant results than other methods while keeping the replication rates comparable.*

*Changes were made in the manuscript lines 261-264, 720-722, and* ***Supplementary Table 3C*** *was added.*

**Supplementary Table 3C:** **Replication results using an additional replication data from Chen et al.**

6.      The GWAS overlap with T1D example needs some more clarifications - SNPs in the HLA region is the most important inherited risk factors for T1D and with no other GWAS studies than the one used by the authors (UKBB) showing association with the particular SNP may indicate this is driven by the major haplotypes.

***Response****: Thank you for pointing this out. We further checked the meQTL – T1D GWAS colocalizations and found that we identified a total of 5 meQTL – T1D GWAS colocalizations in CD8 T-cells (****Supplementary Table 4B****) and 4 of them are in the HLA region (****Supplementary Table 2B****) (the 5 signals were extracted and attached below), indicating that our analyses stress the importance of the HLA region in T1D. The presented one in* ***Figure 4*** *was a visualization example.*

*We also agreed that those colocalized SNPs might be driven by* major *haplotypes/high LD. As colocalization takes both SNP-T1D association (GWAS) and SNP-CpG association (meQTL) into consideration, SNPs having stronger associations in both GWAS and meQTL are more likely to be identified as colocalized/shared SNP (as our example in* ***Figure 4****). However, when examining GWAS data alone, it is possible that these colocalized SNPs are driven by high LD or major haplotypes. For example, one nearby SNP rs9271365 (mapped to HLA-DQA1), which was not among the colocalized list, is identified as the top lead variant associated with T1D in a multi-ancestry GWAS [19]. Similarly, multiple studies identified strong signals for T1D in the HLA region (near the colocalized SNPs identified by us) [20, 21]. These findings suggested that the colocalized signals might be driven by haplotype structures or LD. More clarifications were added to the discussion session.*

*Changes were made in the manuscript lines 425-426, 435-438.*

| Category | Trait 1 (GWAS) | Trait2 (CpG) | Chr | Colocalized SNP | BP for SNP | Nearest gene for SNP |
| --- | --- | --- | --- | --- | --- | --- |
| Immune | Type_1_Diabetes | cg23785275 | 6 | rs9273367 | 32626438 | *HLA-DQB1* |
| Immune | Type_1_Diabetes | cg00886432 | 6 | rs2395178 | 32405362 | *HLA-DRA* |
| Immune | Type_1_Diabetes | cg09059466 | 6 | rs2395360 | 33102536 | *HLA-DPB2* |
| Immune | Type_1_Diabetes | cg07187855 | 6 | rs3130650 | 30818684 | *LINC02570* |
| Immune | Type_1_Diabetes | cg22627029 | 6 | rs3129867 | 32404220 | *HLA-DRA* |

**References**

1.	Schulz H, Ruppert A-K, Herms S, Wolf C, Mirza-Schreiber N, Stegle O, et al. Genome-wide mapping of genetic determinants influencing DNA methylation and gene expression in human hippocampus. Nature Communications. 2017;8(1):1511.

2.	Pierce BL, Tong L, Argos M, Demanelis K, Jasmine F, Rakibuz-Zaman M, et al. Co-occurring expression and methylation QTLs allow detection of common causal variants and shared biological mechanisms. Nature Communications. 2018;9(1):804.

3.	Li B, Aouizerat BE, Cheng Y, Anastos K, Justice AC, Zhao H, et al. Incorporating local ancestry improves identification of ancestry-associated methylation signatures and meQTLs in African Americans. Communications Biology. 2022;5(1):401.

4.	Drong AW, Nicholson G, Hedman AK, Meduri E, Grundberg E, Small KS, et al. The presence of methylation quantitative trait loci indicates a direct genetic influence on the level of DNA methylation in adipose tissue. PLoS One. 2013;8(2):e55923.

5.	Barbeira AN, Bonazzola R, Gamazon ER, Liang Y, Park Y, Kim-Hellmuth S, et al. Exploiting the GTEx resources to decipher the mechanisms at GWAS loci. Genome Biology. 2021;22(1):49.

6.	Houseman EA, Accomando WP, Koestler DC, Christensen BC, Marsit CJ, Nelson HH, et al. DNA methylation arrays as surrogate measures of cell mixture distribution. BMC Bioinformatics. 2012;13(1):86.

7.	Jaffe AE, Irizarry RA. Accounting for cellular heterogeneity is critical in epigenome-wide association studies. Genome Biology. 2014;15(2):R31.

8.	Zhang X, Hu Y, Justice AC, Li B, Wang Z, Zhao H, et al. DNA methylation signatures of illicit drug injection and hepatitis C are associated with HIV frailty. Nature Communications. 2017;8(1):2243.

9.	Liang X, Sinha R, Justice AC, Cohen MH, Aouizerat BE, Xu K. A new monocyte epigenetic clock reveals nonlinear effects of alcohol consumption on biological aging in three independent cohorts (N = 2242). Alcohol Clin Exp Res. 2022;46(5):736-48.

10.	Hannon E, Dempster EL, Davies JP, Chioza B, Blake GET, Burrage J, et al. Quantifying the proportion of different cell types in the human cortex using DNA methylation profiles. BMC Biol. 2024;22(1):17.

11.	Sillanpää MJ, Pikkuhookana P, Abrahamsson S, Knürr T, Fries A, Lerceteau E, et al. Simultaneous estimation of multiple quantitative trait loci and growth curve parameters through hierarchical Bayesian modeling. Heredity. 2012;108(2):134-46.

12.	McRae AF, Marioni RE, Shah S, Yang J, Powell JE, Harris SE, et al. Identification of 55,000 Replicated DNA Methylation QTL. Scientific Reports. 2018;8(1):17605.

13.	Grundberg E, Meduri E, Sandling JK, Hedman AK, Keildson S, Buil A, et al. Global analysis of DNA methylation variation in adipose tissue from twins reveals links to disease-associated variants in distal regulatory elements. Am J Hum Genet. 2013;93(5):876-90.

14.	Pickrell JK, Berisa T, Liu JZ, Ségurel L, Tung JY, Hinds DA. Detection and interpretation of shared genetic influences on 42 human traits. Nat Genet. 2016;48(7):709-17.

15.	Hawe JS, Wilson R, Schmid KT, Zhou L, Lakshmanan LN, Lehne BC, et al. Genetic variation influencing DNA methylation provides insights into molecular mechanisms regulating genomic function. Nature Genetics. 2022;54(1):18-29.

16.	Bennett DA, Buchman AS, Boyle PA, Barnes LL, Wilson RS, Schneider JA. Religious Orders Study and Rush Memory and Aging Project. J Alzheimers Dis. 2018;64(s1):S161-s89.

17.	Agashe C, Chiang D, Grishin A, Masilamani M, Jones SM, Wood RA, et al. Impact of granulocyte contamination on PBMC integrity of shipped blood samples: Implications for multi-center studies monitoring regulatory T cells. J Immunol Methods. 2017;449:23-7.

18.	Chen L, Ge B, Casale FP, Vasquez L, Kwan T, Garrido-Martín D, et al. Genetic Drivers of Epigenetic and Transcriptional Variation in Human Immune Cells. Cell. 2016;167(5):1398-414.e24.

19.	Michalek DA, Tern C, Zhou W, Robertson CC, Farber E, Campolieto P, et al. A multi-ancestry genome-wide association study in type 1 diabetes. Human Molecular Genetics. 2024;33(11):958-68.

20.	Qu H-Q, Qu J, Bradfield J, Marchand L, Glessner J, Chang X, et al. Genetic architecture of type 1 diabetes with low genetic risk score informed by 41 unreported loci. Communications Biology. 2021;4(1):908.

21.	Pociot F. Type 1 diabetes genome-wide association studies: not to be lost in translation. Clin Transl Immunology. 2017;6(12):e162.

**2^nd^ round**

Reviewer #1: The authors have responded to my comments.

Reviewer #2: The authors have responded to my comments and I have no further concerns.
